# Measuring Stimulus-Evoked Neurophysiological Differentiation in Distinct Populations of Neurons in Mouse Visual Cortex

**DOI:** 10.1523/ENEURO.0280-21.2021

**Published:** 2022-02-08

**Authors:** William G. P. Mayner, William Marshall, Yazan N. Billeh, Saurabh R. Gandhi, Shiella Caldejon, Andrew Cho, Fiona Griffin, Nicole Hancock, Sophie Lambert, Eric K. Lee, Jennifer A. Luviano, Kyla Mace, Chelsea Nayan, Thuyanh V. Nguyen, Kat North, Sam Seid, Ali Williford, Chiara Cirelli, Peter A. Groblewski, Jerome Lecoq, Giulio Tononi, Christof Koch, Anton Arkhipov

**Affiliations:** 1 Neuroscience Training Program, University of Wisconsin–Madison, Madison, WI 53705; 2 Department of Psychiatry, University of Wisconsin–Madison, Madison, WI 53719; 3 Department of Mathematics and Statistics, Brock University, St. Catharines, Ontario L2S 3A1, Canada; 4 Allen Institute, Seattle, WA 98109

**Keywords:** calcium imaging, differentiation analysis, perception, population coding

## Abstract

Despite significant progress in understanding neural coding, it remains unclear how the coordinated activity of large populations of neurons relates to what an observer actually perceives. Since neurophysiological differences must underlie differences among percepts, *differentiation analysis*—quantifying distinct patterns of neurophysiological activity—has been proposed as an “inside-out” approach that addresses this question. This methodology contrasts with “outside-in” approaches such as feature tuning and decoding analyses, which are defined in terms of extrinsic experimental variables. Here, we used two-photon calcium imaging in mice of both sexes to systematically survey stimulus-evoked neurophysiological differentiation (ND) in excitatory neuronal populations in layers (L)2/3, L4, and L5 across five visual cortical areas (primary, lateromedial, anterolateral, posteromedial, and anteromedial) in response to naturalistic and phase-scrambled movie stimuli. We find that unscrambled stimuli evoke greater ND than scrambled stimuli specifically in L2/3 of the anterolateral and anteromedial areas, and that this effect is modulated by arousal state and locomotion. By contrast, decoding performance was far above chance and did not vary substantially across areas and layers. Differentiation also differed within the unscrambled stimulus set, suggesting that differentiation analysis may be used to probe the ethological relevance of individual stimuli.

## Significance Statement

Much is known about how neurons encode stimuli in the visual system, yet it remains unclear how their activity generates conscious percepts. Recent studies have linked differentiation of neural activity to subjective ratings of stimulus “meaningfulness” and the presence of consciousness itself. We systematically surveyed different neuronal populations in mouse visual cortex and showed that activity in layers (L)2/3 of the anterolateral and anteromedial areas is more differentiated in response to naturalistic movie stimuli compared with meaningless phase-scrambled stimuli. Contrariwise, decoding performance was high and did not vary substantially across populations. These findings advance our understanding of functional differences among layers and areas and highlight differentiation analysis as a theoretically-motivated approach that can complement analyses that focus on stimulus encoding.

## Introduction

The visual system acts on incoming stimuli to extract meaningful features and guide behavior, a process that transforms physical input into conscious percepts. Since the early experiments of Hubel and Wiesel (1959), neuroscience has yielded considerable insight into the visual system by analyzing neural response properties to uncover which features cells are tuned to and how their activity relates to behavior. Modern decoding approaches have revealed stimulus information in population responses (Quiroga and Panzeri, 2009). However, that a population of neurons represents stimulus information does not imply that this information is used to generate conscious percepts (Brette, 2019). Consequently, despite the success of these “outside-in” methods (Buzsáki, 2019) in understanding neural coding, it remains unclear how the coordinated activity of large neuronal populations relates to what the observer actually sees.

Is there an objective approach that can shed light on this question? *Differentiation analysis*—measuring the extent to which a population of neurons expresses a rich and varied repertoire of states—has been proposed as one such approach (Boly et al., 2015; Mensen et al., 2017, 2018). Differentiation analysis exemplifies “inside-out” methodology in that the spatiotemporal diversity of neural activity (*neurophysiological differentiation*; ND) is quantified without reference to the stimulus or other experimental variables imposed a priori by the investigator, in contrast to feature tuning or decoding analyses.

A visual stimulus can be considered meaningful to the observer if it evokes rich and varied perceptual experiences (*phenomenological differentiation*). For example, an engaging movie is meaningful in this sense, as it evokes many distinct percepts with high-level structure; conversely, flickering “TV noise” essentially evokes a single percept with no high-level structure to a human observer, although, at the level of pixels, any two frames of noise are likely to be more different from each other than a pair of frames from a movie (*stimulus differentiation*; SD). Since conscious percepts are determined by brain states, physical differences must underlie phenomenological differentiation. Thus, one can expect measures of ND to correlate with subjective perception of the “richness” or “meaningfulness” of stimuli to the extent that such measures capture the relevant physical, i.e., neuronal, differences. This has indeed been shown in human studies using fMRI and EEG (Boly et al., 2015; Mensen et al., 2017, 2018). Moreover, integrated information theory (IIT) posits a fundamental relationship between ND and subjective experience itself (Tononi, 2004; Oizumi et al., 2014; Marshall et al., 2016; Tononi et al., 2016), and several studies have shown that loss of ND is implicated in loss of consciousness (Casali et al., 2013; Barttfeld et al., 2015; Hudetz et al., 2015; Wenzel et al., 2019).

Although studies in human subjects suggest that ND can provide a readout of stimulus-evoked phenomenological differentiation (Boly et al., 2015; Mensen et al., 2017, 2018), the low spatial resolution of fMRI and EEG has precluded identifying the cell populations that underlie this correspondence. A longstanding fundamental question is which neuronal populations contribute directly to generating conscious percepts (Koch et al., 2016; Tononi et al., 2016; Mashour et al., 2020). Differentiation analysis may shed light on this question, but to do so, it must be applied to signals from specific populations of neurons.

To address this gap, we used *in vivo* two-photon calcium imaging in mice to measure ND evoked by naturalistic and phase-scrambled movie stimuli in excitatory cell populations in cortical layers (L)2/3, L4, and L5, across five visual cortical areas: primary (V1), lateromedial (LM), anterolateral (AL), posteromedial (PM), and anteromedial (AM). We hypothesized that unscrambled naturalistic stimuli, which presumably elicit meaningful visual percepts, would evoke greater ND than their meaningless phase-scrambled counterparts.

We find that unscrambled stimuli evoke greater ND than scrambled stimuli specifically in L2/3 of areas AL and AM and not in the other neuronal populations. We contrast this layer-specific and area-specific finding with a decoding analysis that shows that information about the stimulus category, whether meaningful or meaningless, is present in most populations. This highlights a key difference: ND is more plausibly correlated with stimulus meaningfulness than the information measured by decoding, since the latter may not be functionally relevant (Brette, 2019). Furthermore, we find differences in evoked ND among the unscrambled stimuli that suggest that differentiation analysis can probe meaningfulness of individual stimuli.

## Materials and Methods

**Figure 1.**
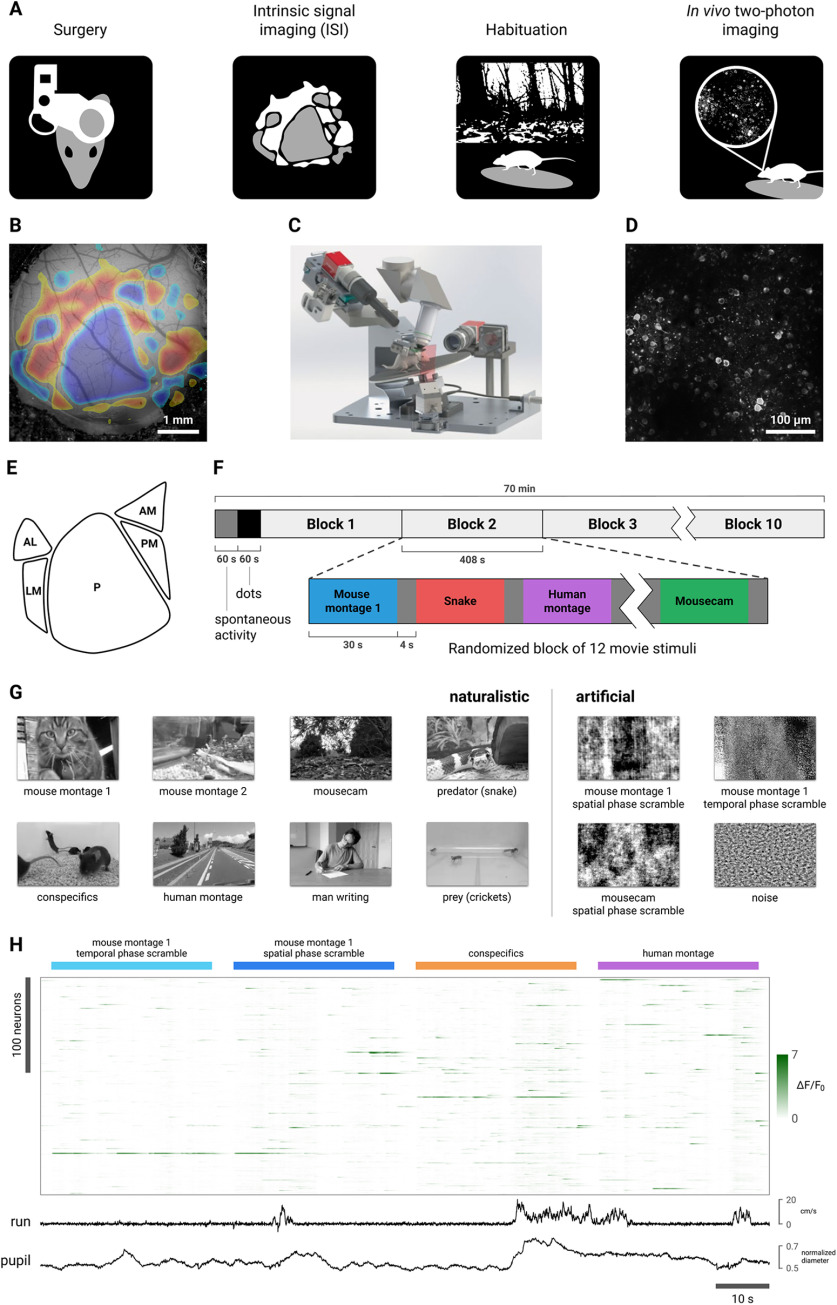
Experimental design. ***A***, Data were acquired using a standardized two-photon calcium imaging pipeline based on that described in de Vries et al. (2020) and Groblewski et al. (2020; Materials and Methods). Briefly, a custom headframe was implanted; ISI was performed to delineate retinotopically mapped visual areas; the mouse was habituated to the passive viewing paradigm over the course of approximately two weeks; and two-photon calcium imaging was performed in the left visual cortex while animals viewed stimuli presented to the contralateral eye in several experimental sessions. ***B***, Example of an ISI map. ***C***, Schematic of the two-photon imaging rig (reproduced with permission from Fig. 1*D* in de Vries et al. 2020). During the imaging sessions, head-fixed mice were free to run on a rotating disk. Locomotion velocity was recorded and pupil diameter was extracted from video of the animal’s right eye. ***D***, Example frame from a two-photon movie. Imaging data were processed as described in de Vries et al. (2020) to obtain ΔF/F_0_ traces. ***E***, Schematic of the five visual areas targeted in this study. ***F***, Ten randomized blocks of 12 30-s movie stimuli were presented; 4 s of mean-luminance gray was presented between stimuli. The first 60-s period was mean-luminance gray (spontaneous activity); the second 60-s period was a high-contrast sparse noise stimulus (not analyzed in this work). ***G***, Still frames from the eight naturalistic (left) and four artificial (right) movie stimuli (see Materials and Methods, Stimuli). Two of the naturalistic stimuli, “mouse montage 1” and “mousecam,” were phase-scrambled to destroy high-level image features while closely matching low-order statistics (see Materials and Methods, Phase scrambling; Extended Data Fig. 1-1). ***H***, Representative calcium imaging and behavioral data. A heatmap of ΔF/F_0_ values is shown for 228 neurons simultaneously imaged in L2/3 of AL during presentation of four stimuli, with locomotion velocity and normalized pupil diameter plotted below. Numbers of cells recorded from each layer and area are listed in Extended Data Figure 1-2. Calcium indicator kinetics did not differ across cell populations (Extended Data Fig. 1-3).

Our experimental design is summarized in Figure 1. We collected calcium imaging data from L2/3, L4, and L5 in each of five visual areas (V1, LM, AL, PM, and AM) across 45 experimental sessions (nine mice, three per layer; L2/3, two males; L4, three males; L5, one male; 15 sessions per transgenic line; 9 sessions per area; 5 ± 1 sessions per mouse; number of cells shown in Extended Data Fig. 1-2). Areas V1, LM, AL, PM, and AM respectively correspond to areas VISp, VISl, VISal, VISpm, and VISam in the Mouse Brain Common Coordinate Framework v3 (Wang et al., 2020). The two-photon calcium imaging pipeline is described in detail in de Vries et al. (2020) and Groblewski et al. (2020). All animal procedures were performed in accordance with the Allen Institute animal care committee’s regulations.

10.1523/ENEURO.0280-21.2021.f1-1 Extended Data Figure 1-1 Stimuli. Twelve 30-s-long greyscale naturalistic (top) and artificial (bottom) movie stimuli were presented. Left, Montages of six 5-s clips. Right, Continuous 30-s clips. Stimuli used in the main analysis are outlined in blue. Arrows indicate the phase-scrambling procedures. Download Figure 1-1, TIF file.

**Figure 2.**
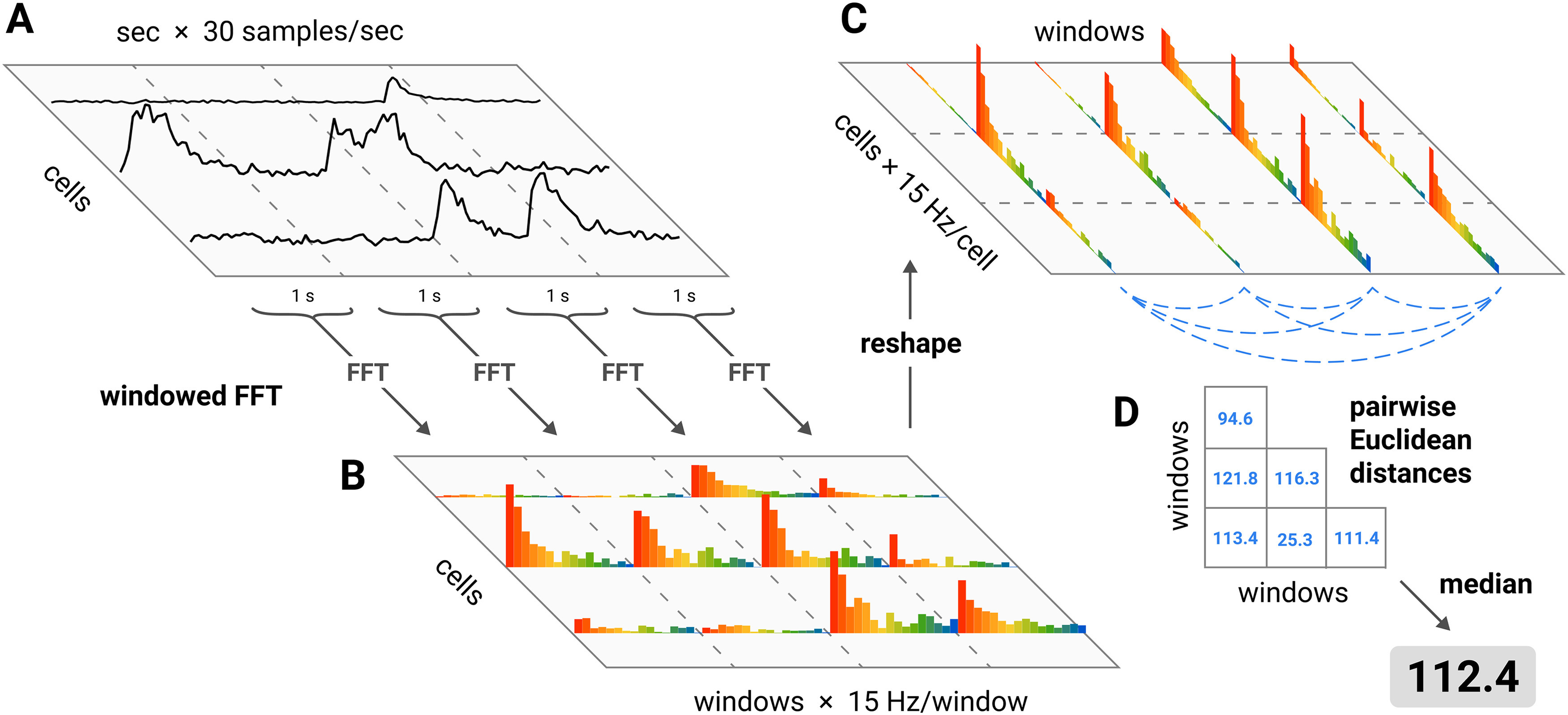
Spectral differentiation analysis. ND was computed as follows. ***A***, For each cell, the ΔF/F_0_ trace during stimulus presentation was divided into 1-s windows. ***B***, The power spectrum of each window was estimated. ***C***, The “neurophysiological state” during each 1-s window was defined as a vector in the high-dimensional space of cells and frequencies (i.e., the concatenation of the power spectra in that window for each cell). ***D***, The ND of the response to a given stimulus was calculated as the median of the pairwise Euclidean distances between every state that occurred during the stimulus presentation. An illustration of how the measure behaves is shown in Extended Data Figure 2-1.

10.1523/ENEURO.0280-21.2021.f1-2 Extended Data Figure 1-2 Number of cells recorded per layer and area. Download Figure 1-2, TIF file.

**Figure 3.**
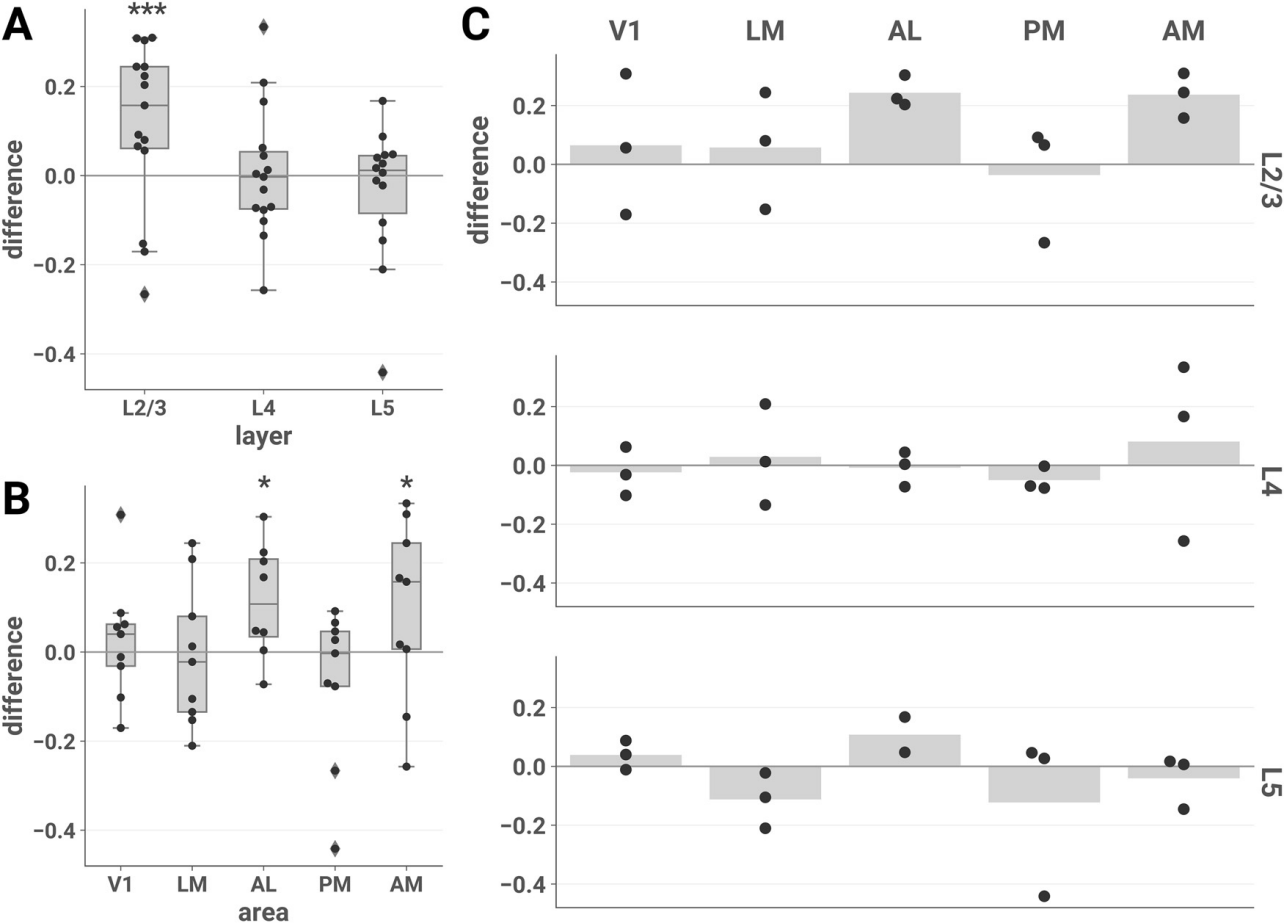
ND elicited by unscrambled versus scrambled stimuli is higher in L2/3 of areas AL and AM. The difference in ND of responses to unscrambled versus scrambled stimuli is plotted for each session by layer (***A***), area (***B***), and layer-area pair (***C***). Each point represents the difference between the mean ND of responses to the two unscrambled and the three scrambled stimuli during a single experimental session. Similar results were found contrasting naturalistic versus artificial stimuli across the entire stimulus set (Extended Data Fig. 3-1). To demonstrate the robustness of this effect, we conducted several further analyses. Sensitivity analyses showed similar findings for various choices of analysis parameters (Extended Data Figs. 3-2, 3-3, 3-4) and when pupil diameter and locomotion were included as covariates in the LME models (Extended Data Figs. 3-5, 3-6, 3-7). We found similar results when we performed the same analysis on discrete calcium events detected from the ΔF/F_0_ traces with an L_0_-regularized algorithm (see Materials and Methods, Event detection), indicating that the effect is driven by differences in the large-timescale patterns of responses rather than small-timescale spectral differences within windows (Extended Data Fig. 3-8). Finally, we also found similar results when we removed event-triggered transients from the ΔF/F_0_ traces, indicating that the effect is not driven solely by initial transients in the calcium response (Extended Data Fig. 3-9). ***A***, ***B***, Asterisks indicate significant *post hoc* one-sided *z*-tests in the layer (***A***) and area (***B***) interaction LME models (*p < 0.05; ***p < 0.001). Boxes indicate quartiles; whiskers indicate the minimum and maximum of data lying within 1.5 times the interquartile range of the 25% or 75% quartiles; diamonds indicate observations outside this range. ***C***, Mean values are indicated by bars.

10.1523/ENEURO.0280-21.2021.f1-3 Extended Data Figure 1-3 Calcium indicator kinetics did not differ across cell populations. Mean (solid line) ± SD (shaded region) calcium response averaged by (***A***) layer and area, (***B***) layer, and (***C***) area. Calcium responses were obtained for each cell by selecting isolated events (those without any other events occurring in the preceding 50 ms or the following 100 ms) and computing the mean event-locked trace (see Materials and Methods, Event detection). ***D***, Responses were well-fit by an exponential decay function; *R*^2^ values of the fit for each cell are plotted by layer and area. ***E***, The fit with the lowest *R*^2^ value, 0.61 (cell 5 in session 718673398, L2/3 AM; fit in red, data in black). We tested for a relationship between layer, area, and response half-life by fitting a LME model with layer, area, and their interaction as fixed effects and experimental session as a random effect and comparing this to a model without the interaction term; we found no layer × area interaction (likelihood ratio test; χ^2^(8) = 1.293, *p* = 0.996). We tested for main effects of layer and area in two further models and likewise found none (layer: χ^2^(2) = 1.143, *p* = 0.565; area: χ^2^(4) = 0.2288, *p* = 0.994). Download Figure 1-3, TIF file.

Our sample size was selected on the basis of a pilot study using existing, publicly available calcium imaging data from the Allen Institute Brain Observatory (de Vries et al., 2020). We measured spectral differentiation of responses to a movie stimulus (clips from the film *Touch of Evil*) versus artificial stimuli (drifting gratings and locally sparse noise) across eight experimental sessions, from which we estimated that we required at least three sessions per layer/area pair to have statistical power of at least 0.8.

### Transgenic mice

We maintained all mice on reverse 12/12 h dark/light cycle following surgery and throughout the duration of the experiment and performed all experiments during the dark cycle. We used the transgenic mouse line *Ai93*, in which GCaMP6f expression is dependent on the activity of both Cre recombinase and the tetracycline controlled transactivator protein (tTA; Madisen et al., 2010). Triple transgenic mice (*Ai93*, *tTA*, *Cre*) were generated by first crossing *Ai93* mice with *Camk2a-tTA* mice, which preferentially express tTA in forebrain excitatory neurons.

*Cux2-CreERT2;Camk2a-tTA;Ai93(TITL-GCaMP6f)* expression is regulated by the tamoxifen-inducible *Cux2* promoter, induction of which results in Cre-mediated expression of GCaMP6f predominantly in superficial cortical L2/3 and L4. *Rorb-IRES2-Cre;Cam2a-tTA;Ai93* exhibit GCaMP6f in excitatory neurons in cortical L4 (dense patches) and L5 and L6 (sparse). Rbp4-Cre;Camk2a-tTA;Ai93 exhibit GCaMP6f in excitatory neurons in cortical L5. Calcium indicator kinetics did not differ between cell populations (Extended Data Fig. 1-3).

### Surgery

Transgenic mice expressing GCaMP6f were weaned and genotyped at ∼P21, and surgery was performed between P37 and P63. The craniotomy was centered at X = –2.8 mm and Y = 1.3 mm with respect to lambda (centered over the left mouse visual cortex). A circular piece of skull 5 mm in diameter was removed, and a durotomy was performed. A coverslip stack (two 5-mm and one 7-mm glass coverslips adhered together) was cemented in place with Vetbond. Metabond cement was applied around the cranial window inside the well to secure the glass window.

### Intrinsic imaging

To define area boundaries and target *in vivo* two-photon calcium imaging experiments to consistent retinotopic locations, retinotopic maps for each animal were created using intrinsic signal imaging (ISI) while mice were lightly anesthetized with 1–1.4% isoflurane. This procedure and data processing pipeline are described in detail in de Vries et al. (2020).

### Habituation

Following successful ISI mapping, mice spent two weeks being habituated to head fixation and visual stimulation. During the second week, mice were head-fixed and presented with visual stimuli, starting with 10 min and progressing to 50 min of visual stimuli by the end of the week. During this week they were exposed to the “mouse montage 2” stimulus (see below, Stimuli).

### Imaging

Calcium imaging was performed using a two-photon-imaging instrument (Nikon A1R MP+). Laser excitation was provided by a Ti:Sapphire laser (Chameleon Vision – Coherent) at 910 nm. Mice were head-fixed on top of a rotating disk and free to run at will. The screen center was positioned 118.6 mm lateral, 86.2 mm anterior, and 31.6 mm dorsal to the right eye. The distance between the screen and the eye was 15 cm. Movies were recorded at 30 Hz using resonant scanners over a 400-μm field of view.

### Locomotion

Locomotion velocity was recorded from the running wheel and preprocessed as follows. First, artifacts were removed using custom code that iteratively identified large positive or negative peaks (indicative of artifactual discontinuities in the signal) in several passes of scipy.signal.find_peaks (specific parameters were manually chosen for each session). Remaining artifacts were then manually removed by inspecting the resulting timeseries and visually identifying clear discontinuities. The removed samples were filled using linear interpolation (pandas.Series.interpolate).

The resulting signal was then low-pass filtered at 1 Hz using a zero-phase fourth-order Butterworth filter [scipy.signal.butter(2, 1/15, btype='lowpass', output='ba', analog=False) applied with scipy.signal.filtfilt].

For the effect size analysis, the fraction of time spent running was calculated by binarizing the preprocessed velocity timeseries at a threshold of 2.5 cm/s.

### Pupillometry

Pupil diameter was extracted from video of the mouse’s ipsilateral eye (relative to the stimulus presentation monitor) using the AllenSDK (https://github.com/AllenInstitute/AllenSDK) as described in de Vries et al. (2020).

Briefly, for each frame of the video an ellipse was fitted to the region corresponding to the pupil as follows: a seed point within the pupil was identified via convolution with a black square; 18 rays were drawn starting at this seed point, spaced 20° apart; the candidate boundary point between the pupil and iris along that ray was identified by a change in pixel intensity above a session-specific threshold; a RANSAC algorithm was used to fit the an ellipse to the candidate boundary points using linear regression with a conic section constraint; and fitted parameters of the regression were converted to ellipse parameters (coordinates of the center, lengths of the semi-major and semi-minor axes, and angle of rotation with respect to the *x*-axis). Pupil diameter was taken to be twice the semi-major axis of the fitted ellipse.

The resulting timeseries contained some artifacts, which we removed by the same combination of automated and manual methods used for the locomotion timeseries (see above, Locomotion). Each pupil diameter timeseries was then normalized by dividing by the maximum diameter that occurred within the 10 blocks of stimulus presentations during that session.

### Event detection

Discrete calcium events were detected from the ΔF/F_0_ traces using the L_0_-penalized method of Jewell and Witten (2018) and Jewell et al. (2020). This procedure, which replaces the continuous relative changes in fluorescence with discrete, real valued events, is described in detail in de Vries et al. (2020); code is available at https://github.com/AllenInstitute/visual_coding_2p_analysis/blob/master/visual_coding_2p_analysis/l0_analysis.py.

### Stimuli

We created twelve 30-s greyscale naturalistic and artificial movie stimuli.

The eight naturalistic stimuli (Extended Data Fig. 1-1, top) consisted of three montages of six 5-s clips, spliced together with jump cuts, and four continuous stimuli. The “mouse montage 1” stimulus contained clips of conspecifics, a snake, movement at ground level through the underbrush of a wooded environment, and a cat approaching the camera. The “mouse montage 2” stimulus contained different footage of movement through the wooded environment; different footage of a cat approaching the camera; conspecifics in a home cage filmed from within the cage; crickets in a home cage filmed from within the cage; footage of the interior of the home cage with environmental enrichment (a shelter, running wheel, and nesting material); and a snake filmed at close range orienting toward the camera. The “human montage” contained clips of a man talking animatedly to an off-screen interviewer; a café table where food is being served; automobile traffic on a road viewed from above; a woman in the foreground taking a photograph of a city skyline; footage of a road filmed from the passenger seat of a vehicle; and a close shot of a bowl of fruit being tossed. The four continuous stimuli were: footage of a snake at close range orienting toward the camera; crickets in a home cage filmed from within the cage; a man writing at a table; movement through a wooded environment at ground level; and conspecifics in a home cage. No two stimuli contained identical clips.

The four artificial stimuli (Extended Data Fig. 1-1, bottom) consisted of two phase-scrambled versions of the “mouse montage 1” stimulus, a phase-scrambled version of the “mousecam” stimulus (see below, Phase scrambling), and a high-pass-filtered 1/*f* noise stimulus.

Stimuli were presented in a randomized block design with 10 repetitions, with 4 s of static mean-luminance gray presented between stimuli (Fig. 1*F*). 60 s of mean-luminance gray (to record spontaneous activity) and a 60-s high-contrast sparse noise stimulus were also presented in the beginning of each session (not analyzed in this work).

### Phase scrambling

Two methods of phase scrambling were used: temporal and spatial, described in detail below. Briefly, for the temporal scrambling we independently randomized the phase of each pixel’s intensity timeseries in contiguous, nonoverlapping windows of 1 s. For the spatial scrambling, we randomized the phase of the spatial dimensions of the three-dimensional spectrum of each window. The “mouse montage 1” stimulus was phase-scrambled using both procedures to obtain the “mouse montage 1, temporal phase scramble” and “mouse montage 1, spatial phase scramble” stimuli. The “mousecam” stimulus was scrambled using the spatial procedure to obtain the “mousecam, spatial phase scramble” stimulus.

#### Temporal phase scramble

First, the stimuli were windowed into contiguous, nonoverlapping 1-s segments (30 frames each). For each 1-s window, we applied the following procedure:

We estimated the one-dimensional spectrum of each pixel’s intensity timeseries with the discrete Fourier transform (DFT) using the NumPy function numpy.fft.fft. The phase and magnitude of each spectrum were computed with numpy.angle and numpy.abs, respectively. For each pixel, we generated a 14-element random vector drawn uniformly from the interval [0, 2π]. A randomized phase was then obtained for that pixel by concatenating the first element of the original phase, the random vector, the 15th element of the original phase, and the negative reversed random vector. This yielded a 30-element phase vector with the required conjugate symmetry of the spectrum of a 1-s real-valued signal sampled at 30 frames per second. The randomized phase was then combined with the spectral magnitude and transformed back into the time domain with the inverse DFT using numpy.fft.ifft, yielding a temporally phase-scrambled version of that pixel’s intensity timeseries. Each pixel’s timeseries was independently phase-scrambled in this fashion.

This resulted in 30 independently phase-scrambled 1-s windows. These windows were then concatenated to obtain the full 30-s temporally phase-scrambled stimulus.

#### Spatial phase scramble

First, the stimuli were windowed into contiguous, nonoverlapping 1-s segments (30 frames each). For each window, we applied the following procedure. The three-dimensional Fourier spectrum (frame, width, and height) was estimated with the DFT using numpy.fft.fftn. The phase and magnitude of the spectrum were computed with numpy.angle and numpy.abs, respectively. To randomize the phase in the spatial dimensions, we generated a random signal in the time domain with the same dimensions as a stimulus frame (192 pixels wide by 120 pixels high) and computed its phase in the frequency domain as described above. This two-dimensional random spatial phase was added to the spatial dimensions of the three-dimensional stimulus phase. After being randomized in this way, the stimulus phase was recombined with the spectral magnitude and transformed back into a time-domain signal with the inverse DFT using numpy.fft.ifftn. The 30 resulting phase-scrambled 1-s windows were then concatenated to obtain the full 30-s spatially phase-scrambled stimulus.

#### Effect of phase scrambling

The greyscale movie stimuli were represented in the stimulus presentation software as arrays of unsigned eight-bit integers. The limitations of this representation resulted in phase-scrambled stimuli with power spectra that were close but not identical to the power spectrum of their unscrambled counterparts.

Specifically, although the phase scrambling procedures described above leave the power spectrum unchanged, they do not necessarily preserve the range of the resulting real-valued signal. In our case, applying these procedures to our stimuli resulted in phase-scrambled stimuli in which the pixel intensities occasionally lay outside the range [0, 255]. Thus, to represent the phase-scrambled stimuli with eight-bit integers, we truncated the result so that negative intensities were set to 0 and intensities >255 were set to 255. This operation does affect the power spectra, and as a result the spectra of the unscrambled and scrambled stimuli are closely matched but not equal.

### Differentiation analysis

#### Spectral differentiation

Our analysis of the responses to the stimuli follows the techniques developed in previous work in humans (Boly et al., 2015; Mensen et al., 2017, 2018). The spectral differentiation measure of ND used by Mensen et al. (2018) was designed for analysis of timeseries responses to continuous movie stimuli, and was found to be positively correlated with subjective reports of stimulus “meaningfulness.” We employed this measure with our calcium imaging data on single-trial responses: (1) the ΔF/F_0_ trace of each cell during stimulus presentation was divided into 1-s windows; (2) the power spectrum of each window was estimated using a Fourier transform; (3) the “neurophysiological state” during each 1-s window was defined as a vector in the high-dimensional space of cells and frequencies (i.e., the concatenation of the power spectra in that window for each cell); (4) the ND in response to a given stimulus was calculated as the median of the pairwise Euclidean distances between every state that occurred during the stimulus presentation. A schematic illustration is shown in Figure 2, and an illustration of how the measure behaves for different types of signals is shown in Extended Data Figure 2-1.

10.1523/ENEURO.0280-21.2021.f2-1 Extended Data Figure 2-1 Differentiation for simulated signals. To illustrate how the ND measure behaves, we generated three artificial signals and computed ND for each. Signals were normalized to have the same energy. ***A***, ***B***, Artificial spike trains were convolved with an idealized GCaMP6f response kernel (difference of exponentials; decay time constant 0.6 s, rise time constant 0.05 s; Chen et al., 2013; Pachitariu et al., 2018) and downsampled to 30 Hz. ***A***, Periodic bursting at 1 Hz. Because the period is the same as the window length used in the spectral estimation step (Fig. 2*A*), the estimated spectrum of each window is identical, and differentiation is zero. ***B***, An irregular firing pattern has high differentiation. ***C***, Gaussian noise. The theoretical spectrum is identical for each window, but differentiation is nonzero due to the spectral estimation error resulting from the finite window length. Download Figure 2-1, TIF file.

The relationship between action potentials and the resulting calcium imaging signal is complex (Chen et al., 2013; Deneux et al., 2016; Pachitariu et al., 2018; Ledochowitsch et al., 2019; Wei et al., 2020; Huang et al., 2021; Siegle et al., 2021a). The ΔF/F_0_ signal approximately represents a convolution of the underlying spike train with the calcium-dependent fluorescence response kernel, which depends nonlinearly on the spike rate. A consequence of the nonlinearity is that calcium imaging is much more sensitive to burst-like activity than to isolated spikes. Since this convolution affects the spectral properties of the signal, some discussion of its impact on the spectral differentiation measure is warranted. The energy of the GCaMP6f response is concentrated in low frequencies, so for our purposes, the effect of the convolution is that differences among the spectral states are amplified at lower frequencies and attenuated at higher frequencies. Thus, when applied to the ΔF/F_0_ signal, the measure will be less sensitive to short-timescale differences between activity patterns than it would if it were applied directly to the ground-truth spike train. This is not necessarily a disadvantage, as our aim in using a spectral measure in the first place was to achieve temporal smoothing to detect differences in temporal structure on the scale of the state window size.

We normalized spectral differentiation values by the square root of the number of cells in the recorded population, reasoning as follows. Consider a hypothetical population of cells that each exhibit the same temporal pattern of activity. The spectral differentiation of such a population will be proportional to the square root of its size because the Euclidean distance is used to compare neurophysiological states. If we have two such populations differing only in the number of cells, their activity should be considered equally differentiated for our purposes, since their temporal patterns are identical; any differences in spectral differentiation would be due to the (arbitrary) number of cells captured in the imaging session. Thus, we divided by the square root of the population size to remove this dependency.

To investigate the properties of the signal that drive differences in spectral differentiation, we applied the measure to discrete L_0_ calcium events detected from the ΔF/F_0_ traces (see above, Event detection) and obtained similar results as in the main analysis (Extended Data Fig. 3-8). This indicates that the observed differences in spectral differentiation are driven by differences in the large-timescale patterns of responses rather than small-timescale spectral differences within the windows, consistent with the sparsity of calcium responses in this dataset. We also measured ND of ΔF/F_0_ traces with transients removed. Transients were defined as the 200 ms (six imaging samples) following a L_0_ calcium event. This analysis yielded similar results as well (Extended Data Fig. 3-9), indicating that ND differences are not driven solely by initial transients in the calcium response.

For the analysis of SD (Fig. 8), stimuli were first blurred with a circular Gaussian filter whose half width at half maximum was set to the median radius of a L2/3 V1 receptive field (RF) as measured by de Vries et al. (2020; 8.92°) to account for the coarseness of mouse vision. SD was then calculated by treating each pixel of the stimulus as a “cell” and applying the spectral differentiation measure to the traces of pixel intensities over time.

#### Multivariate differentiation

We also measured ND using a multivariate approach that considers spatiotemporal differences in activity patterns. For each experimental session, we selected ΔF/F_0_ traces recorded during presentations of unscrambled stimuli and their scrambled counterparts and concatenated them to obtain an *m *×* n* matrix of responses, where *m* is the number of two-photon imaging samples and *n* is the number of traces. We used a nonlinear dimensionality reduction procedure, Uniform Manifold Approximation and Projection for Dimension Reduction (Python package umap-learn; McInnes et al., 2020), to reduce this matrix to *m *×* *8 with parameters UMAP(n_components = 8, metric=“euclidean”, n_neighbors = 50, min_dist = 0.5). Each row of the resulting matrix was an 8-dimensional vector that represented the state of the cell population during the corresponding two-photon sample. We then grouped the rows of the resulting matrix by stimulus presentation. Each row vector can be thought of as a point in ${\mathbb{R}}^{8}$, so that each trial was associated with a cloud of points corresponding to the population states that the stimulus evoked during that presentation.

The intuition motivating this approach is that we can operationalize the notion of ND by measuring the dispersion of this point cloud. The more distant two points are, the more different are the corresponding responses of the cell population; thus, if a stimulus evokes many different population states, the point cloud will be more spread out in response space. Therefore, we measured ND evoked during each stimulus presentation by finding the centroid of the associated point cloud and taking the mean Euclidean distance of each point to the centroid.

In the multivariate differentiation analysis of calcium events, population response vectors were obtained by summing event magnitudes within 1-s bins to match the definition of the neurophysiological state of the population used in the spectral differentiation analyses. Because the event data were sparse and because including many duplicate instances of the zero vector will reduce the sensitivity of the multivariate differentiation measure to differences among bins in which the population was active, bins with no events were discarded before the dimensionality reduction step.

### Statistical analyses

All analyses were performed with custom Python and R code, using numpy (Harris et al., 2020), scipy (Virtanen et al., 2020), pandas (Reback, 2020), scikit-learn (Pedregosa et al., 2011), matplotlib (Hunter, 2007), seaborn (Waskom, 2020), lme4 (Bates et al., 2015), multcomp (Hothorn et al., 2008), and emmeans (Lenth, 2020). See Table 2 for distributions of data, types of statistical test used, and confidence intervals.

#### Linear mixed effects (LME) models

For analysis of ND across all experimental sessions (Figs. 3, 5; Extended Data Figs. 3-1, 3-8, 3-9, 5-1), we employed LME models using the lmer function from the lme4 package in R with REML = FALSE (Bates et al., 2015). The distributions of ND values for both spectral and multivariate differentiation measures were well-approximated by log-normal distributions, so we applied a logarithmic transformation to ND values before statistical modeling.

10.1523/ENEURO.0280-21.2021.f3-1 Extended Data Figure 3-1 Naturalistic versus artificial differences in ND across the entire stimulus set. The mean difference in ND of responses to all eight naturalistic versus all four artificial stimuli is plotted for each session by layer (***A***), area (***B***), and layer-area pair (***C***). Results are similar to the unscrambled versus scrambled contrast shown in Figure 3. In this analysis, *post hoc* tests showed a significant effect also in L5; however, this contrast does not control for low-level stimulus characteristics and is thus harder to interpret. ***A***, We fit an LME model with stimulus category (naturalistic or artificial), layer, and their interaction as fixed effects and found a significant interaction (likelihood ratio test, χ^2^(2) = 16.343, *p* = 2.83e–4). *Post hoc* one-sided *z*-tests (adjusted for multiple comparisons): L2/3, *z *=* *4.974, *p* = 9.82e–7, Cohen’s *d *=* *0.153, 95% CI [0.064, ∞); L4, *z *= –0.450, *p* = 0.965, Cohen’s *d* = –0.019, 95% CI [–0.057, ∞); L5, *z *=* *3.745, *p* = 2.71e–4, Cohen’s *d *=* *0.144, 95% CI [0.037, ∞). ***B***, We fit an LME model with stimulus category (naturalistic or artificial), area, and their interaction as fixed effects and found a significant interaction (likelihood ratio test, χ^2^(2) = 16.343, *p* = 0.000283). *Post hoc* one-sided *z*-tests (adjusted for multiple comparisons): V1, *z *=* *1.207, *p* = 0.453, Cohen’s *d *=* *0.066, 95% CI [–0.032, ∞); LM, *z *=* *1.523, *p* = 0.281, Cohen’s *d *=* *0.074, 95% CI [–0.023, ∞); AL, *z *=* *4.715, *p* = 6.04e–6, Cohen’s *d *=* *0.222, 95% CI [0.073, ∞); PM, *z *= –0.907, *p* > 0.999, Cohen’s *d* = –0.040, 95% CI [–0.093, ∞); AM, *z *=* *4.249, *p* = 5.37e–05, Cohen’s *d *=* *0.156, 95% CI [0.056, ∞). ***A***, ***B***, Asterisks indicate significant *post hoc* tests in the layer (***A***) and area (***B***) interaction LME models (***p < 0.001). Boxes indicate quartiles; whiskers indicate the minimum and maximum of data lying within 1.5 times the interquartile range of the 25% or 75% quartiles; diamonds indicate observations outside this range. ***C***, Mean values are indicated by bars. Download Figure 3-1, TIF file.

10.1523/ENEURO.0280-21.2021.f3-2 Extended Data Figure 3-2 Sensitivity analysis of main ND results. We investigated the sensitivity of our results to changes in various parameters of the ND calculation. We systematically varied (1) the distance metric used to compare population state vectors (vertical axis of heatmaps); (2) the length of the window that defines a single state, in which the spectrum is estimated (horizontal axis of heatmaps); (3) the spacing of the frequency bins in the estimated spectrum (linear, ***A***, ***B***; logarithmic, ***C***, ***D***); and (4) the window type and amount of overlap used in estimating the spectra across the stimulus presentation (shown in the following two figures). For each combination of these parameters, we computed ND values and performed the same statistical analysis as described in the main text. Each cell in the heatmaps in ***A***, ***C*** shows the *p* value of the likelihood ratio test for the stimulus category × layer interaction (left) and stimulus category × area interaction (right); cells in ***B***, ***D*** show *p* values for the associated *post hoc* tests. The results reported in the main text correspond to the second row and third column of the heatmaps in ***A***, ***B***. For nearly all other combinations of parameters, we likewise find that unscrambled stimuli elicit increased ND specifically in L2/3 of AL and AM. Download Figure 3-2, TIF file.

10.1523/ENEURO.0280-21.2021.f3-3 Extended Data Figure 3-3 Sensitivity analysis was performed as described in Extended Data Figure 3-2, except that the time-frequency analysis step of computing ND (Fig. 2*A*) was performed using a Tukey window with 12.5% overlap. Download Figure 3-3, TIF file.

**Figure 4.**
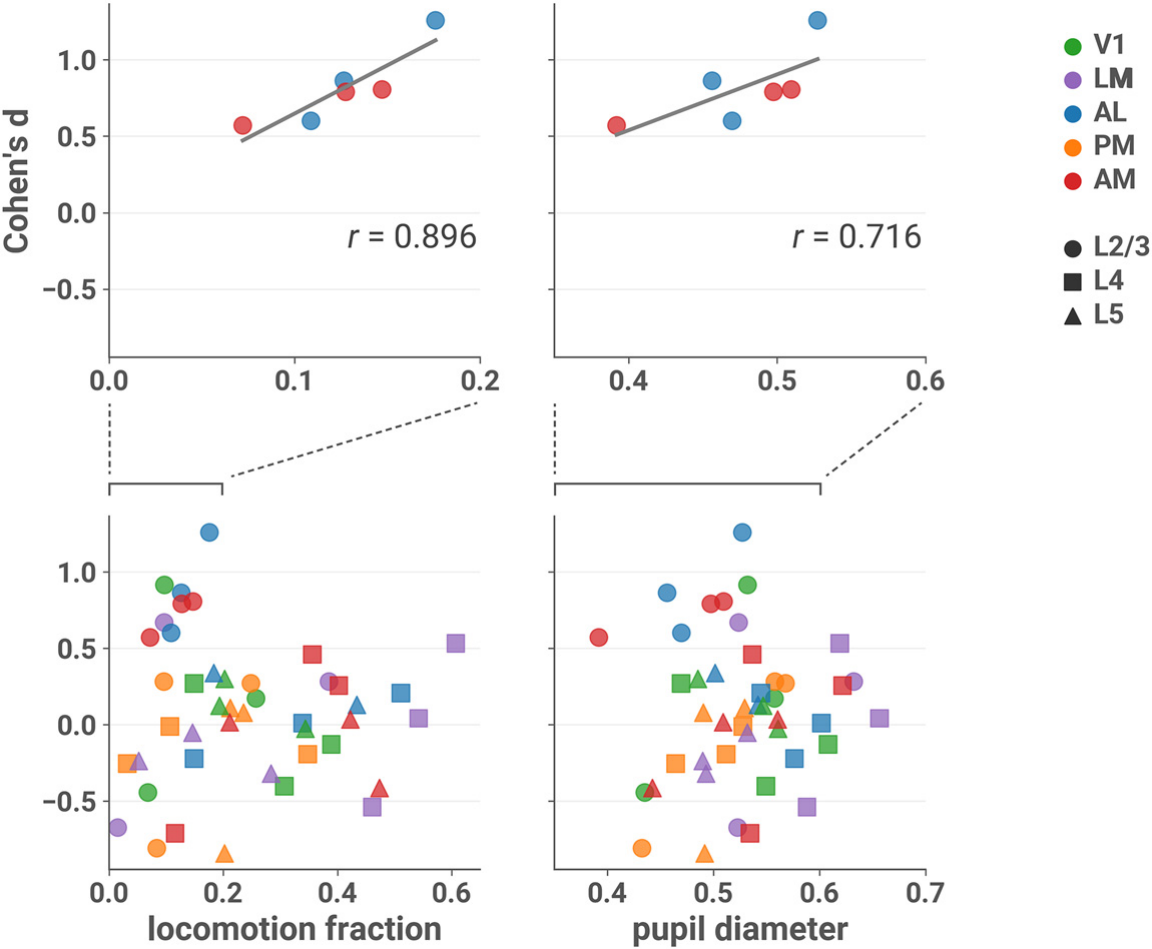
Effect sizes in L2/3 of AL and AM are larger in sessions with more locomotion and larger pupil diameter. Cohen’s *d* is plotted against the fraction of locomotion activity (left column) and mean normalized pupil diameter (right column) during the session, with linear fit in gray. Top row: only sessions recorded from L2/3 and areas AL or AM. Bottom row: all sessions (note different scales). Top left: Pearson’s *r *=* *0.896 (two-sided *t* test; *t*_(4)_ = 4.030, *p* = 0.0157, 95% CI [0.308, 1.00]^l^). Top right: *r *=* *0.716 (*t*_(4)_ = 2.054, *p* = 0.109, 95% CI [–0.227, 1.00]^m^). Running velocity >2.5 cm/s was considered locomotion activity (see Materials and Methods, Locomotion). Normalized pupil diameter was obtained by dividing by the maximum diameter that occurred during the session (see Materials and Methods, Pupillometry).

10.1523/ENEURO.0280-21.2021.f3-4 Extended Data Figure 3-4 Sensitivity analysis was performed as described in Extended Data Figure 3-2, except with a Kaiser window (β = 14) with 50% overlap. Download Figure 3-4, TIF file.

**Figure 5.**
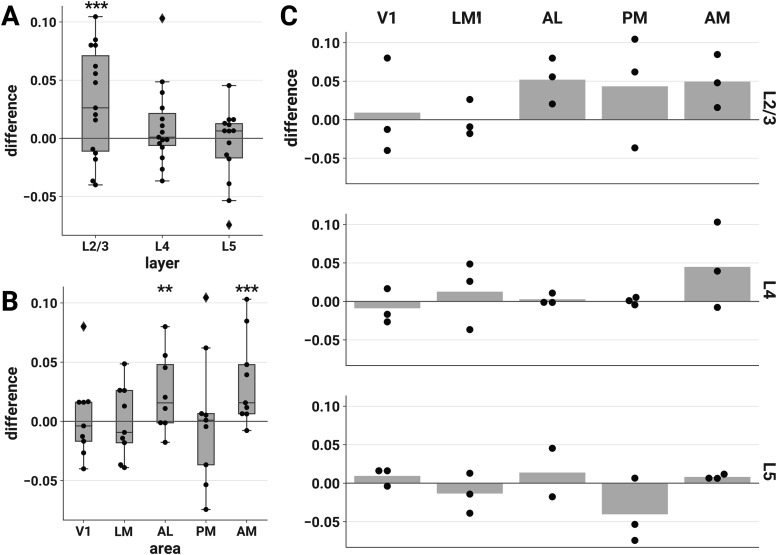
Multivariate differentiation analysis. The mean difference in the mean centroid distance of responses to unscrambled versus scrambled stimuli is plotted for each session by layer (***A***), area (***B***), and layer-area pair (***C***). ND elicited by unscrambled versus scrambled stimuli is higher in L2/3 and areas AL and AM, consistent with the spectral differentiation analysis. We found similar results when we analyzed discrete L_0_ calcium events detected from the ΔF/F_0_ traces (see Materials and Methods, Event detection; Extended Data Fig. 5-1). ***A***, ***B***, Asterisks indicate significant *post hoc* one-sided *z*-tests in the layer (***A***) and area (***B***) interaction LME models (**p < 0.01; ***p < 0.001). Boxes indicate quartiles; whiskers indicate the minimum and maximum of data lying within 1.5 times the interquartile range of the 25% or 75% quartiles; diamonds indicate observations outside this range. ***C***, Mean values are indicated by bars.

10.1523/ENEURO.0280-21.2021.f3-5 Extended Data Figure 3-5 Sensitivity analysis for LME models including arousal variables (locomotion and pupil diameter) as covariates. Consistent with results from the simpler models, L2/3 of AL and AM emerge as the cell populations in which ND is greater for unscrambled versus scrambled stimuli for nearly all parameter combinations. Download Figure 3-5, TIF file.

**Figure 6.**
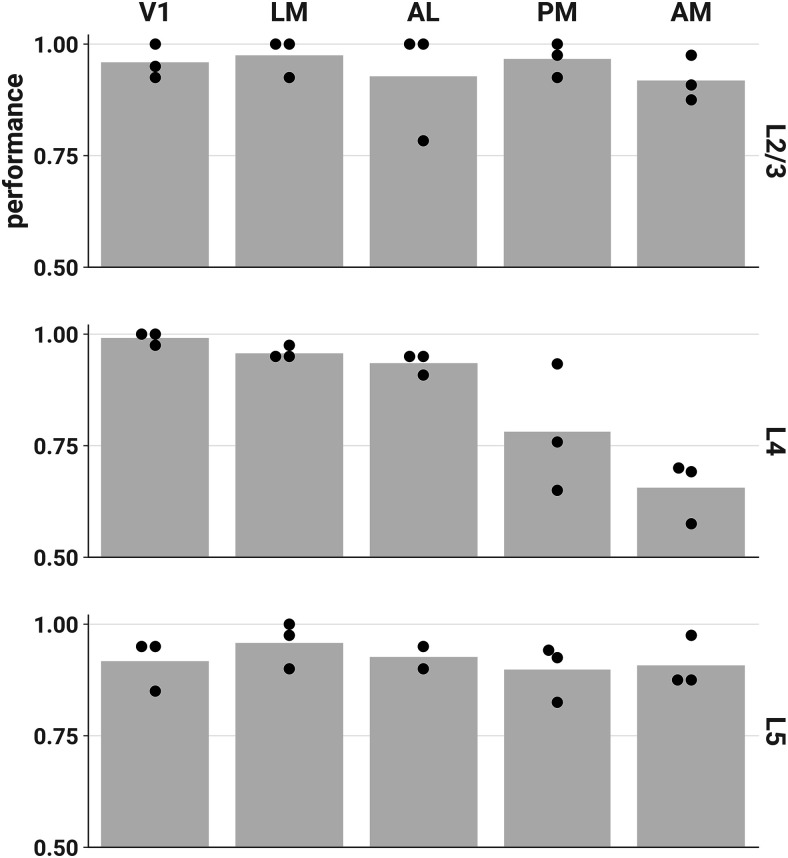
Stimulus category (unscrambled or scrambled) can be accurately decoded from most layers and areas. Each point represents the mean fivefold cross-validated balanced accuracy score of linear discriminant analysis performed on a single session (see Materials and Methods, Decoding analyses). Chance performance is 0.5. We found similar results when decoding stimulus identity across all 12 stimuli (Extended Data Fig. 6-1).

10.1523/ENEURO.0280-21.2021.f3-6 Extended Data Figure 3-6 Sensitivity analysis as in Extended Data Figure 3-5, using a Tukey window with 12.5% overlap. Download Figure 3-6, TIF file.

**Figure 7.**
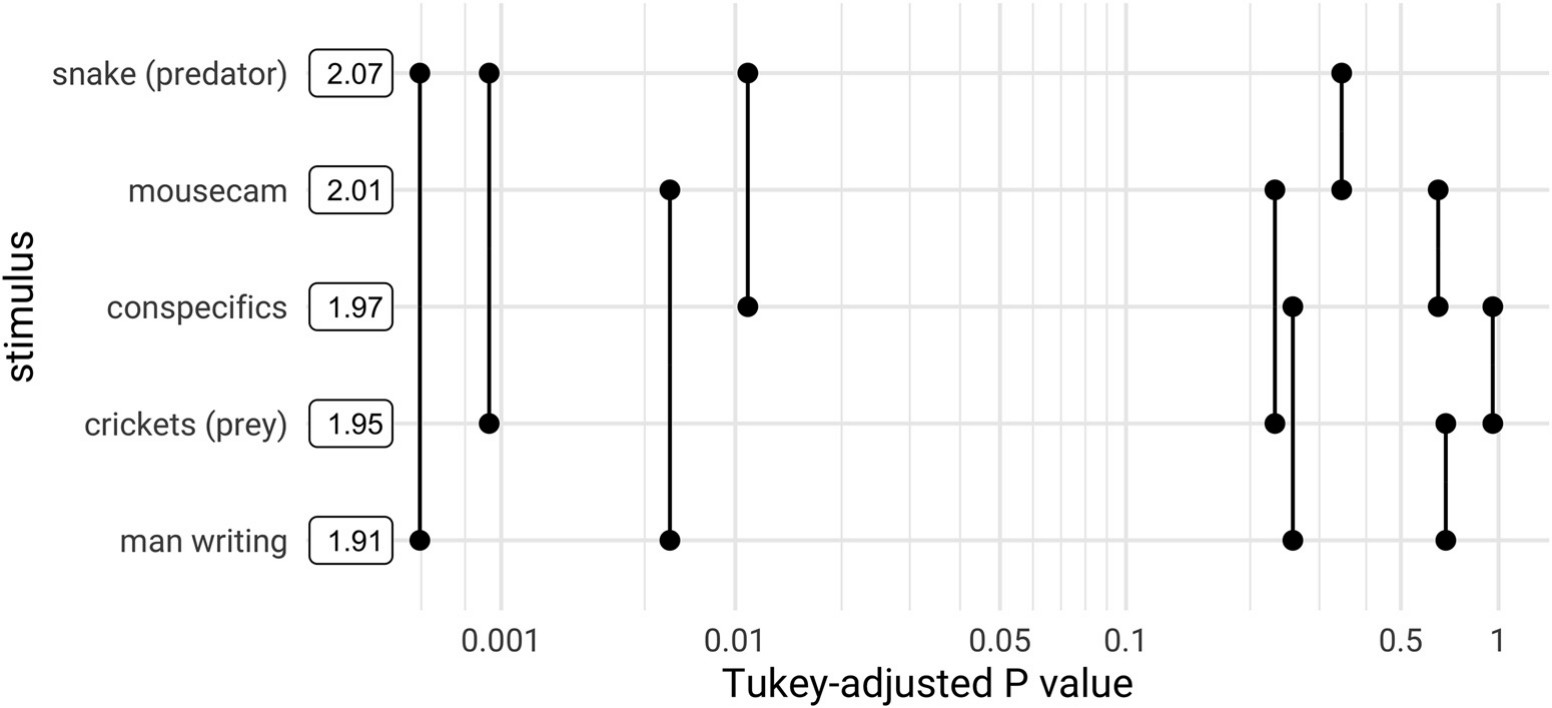
Pairwise differences in ND among unscrambled, continuous stimuli. *Post hoc* pairwise comparisons using data from all neuronal populations are plotted against their *p* values (adjusted for multiple comparisons). Boxes show mean ND for each stimulus. ND of the “snake (predator)” stimulus is significantly greater than that of “crickets” and “man writing” at a threshold of α = 0.01, and greater than “conspecifics” at α = 0.05. ND of the “mousecam” stimulus is greater than that of “man writing” at α = 0.01. Mediation analysis showed a mixture of direct and arousal-mediated effects, indicating that changes in arousal cannot fully account for these differences (Extended Data Fig. 7-1). Pairwise differences in ND and decoding performance stratified by layer and area are shown in Extended Data Figure 7-2.

10.1523/ENEURO.0280-21.2021.f3-7 Extended Data Figure 3-7 Sensitivity analysis as in Extended Data Figure 3-5, using a Kaiser window (β = 14) with 50% overlap. Download Figure 3-7, TIF file.

**Figure 8.**
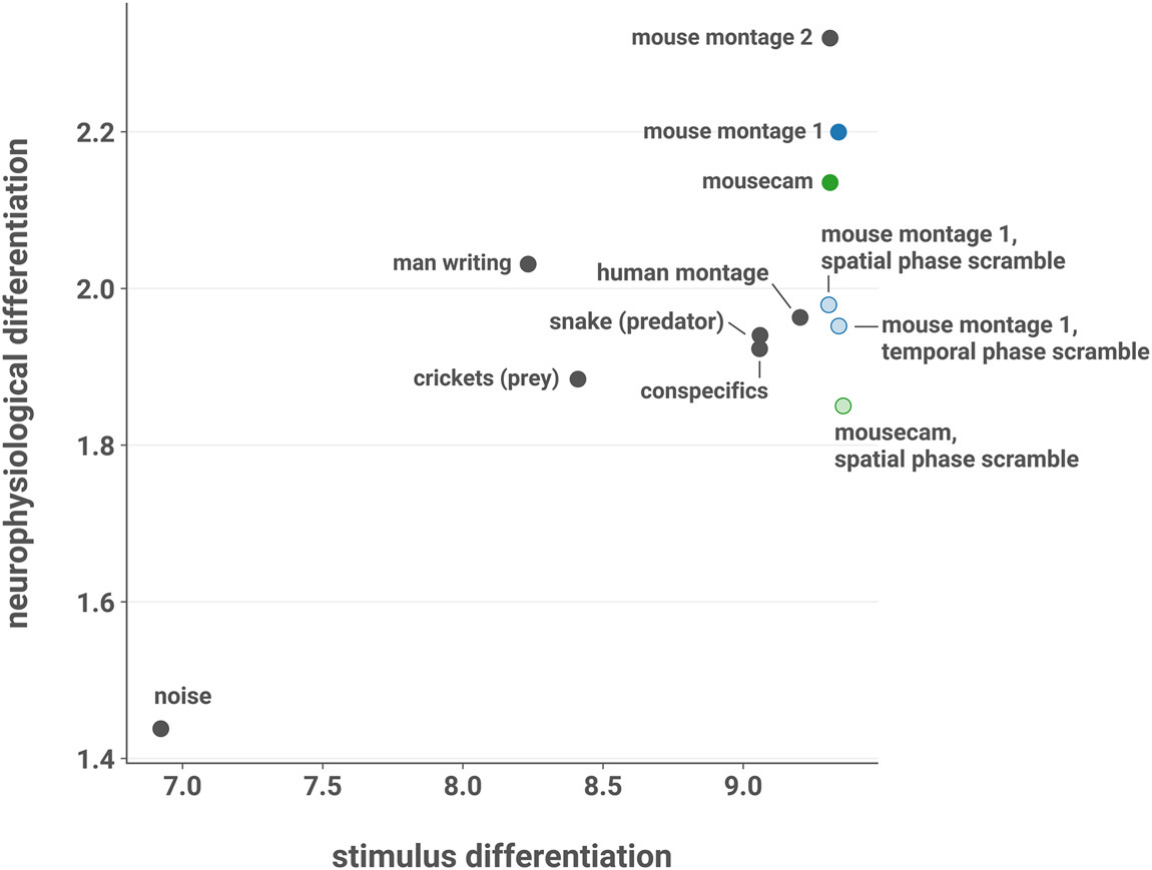
SD does not explain ND. Mean ND elicited by each stimulus in L2/3 of AL and AM, plotted against SD. SD was computed by treating each pixel of the movie as a “cell” and applying the spectral differentiation measure to traces of pixel intensities over time after blurring the movie with a Gaussian filter to account for the coarseness of mouse vision. Across all stimuli, mean ND is positively correlated with SD (Pearson’s *r *=* *0.746; one-sided *t* test; *t*_(10)_ = 3.542, *p* = 0.00267, 95% CI [0.393, 1.00]^hh^). However, here the noise stimulus is a highly influential observation (Cook’s *D *=* *2.318, an order of magnitude larger than the next most influential observation). With the noise stimulus excluded, the correlation is weaker (*r = *0.258; one-sided *t* test; *t*_(9)_ = 0.801, *p* = 0.222, 95% CI [–0.307, 1.00]^ii^). Moreover, there was no evidence of a relationship with ND when considering only the scrambled stimuli and their unscrambled counterparts (*r* = –0.378; two-sided *t* test; *t*_(3)_ = –0.708*, p* *=* 0.523, 95% CI [–0.945, 0.756]^jj^). ND was also not explained by variation in stimulus luminance, contrast, or spectral energy (Extended Data Fig. 8-1).

10.1523/ENEURO.0280-21.2021.f3-8 Extended Data Figure 3-8 Spectral differentiation analysis of discrete L_0_ calcium events. The difference in ND of responses to unscrambled versus scrambled stimuli is plotted for each session by layer (***A***), area (***B***), and layer-area pair (***C***). Results are similar to the main analysis on ΔF/F_0_ traces shown in Figure 3. This indicates that the differences we observed in ND are driven by differences in the large-timescale patterns of responses rather than small-timescale spectral differences within the windows, consistent with the sparsity of calcium responses in our dataset. ***A***, We fit an LME model with stimulus category (naturalistic or artificial), layer, and their interaction as fixed effects and found a significant interaction (likelihood ratio test, χ^2^(2) = 11.481, *p* = 0.00321). *Post hoc* one-sided *z*-tests (adjusted for multiple comparisons): L2/3, *z *= 3.835, *p* = 0.000188, Cohen’s *d* = 0.175, 95% CI [0.0478, ∞); L4, *z *= –0.154, *p* = 0.916, Cohen’s *d* = –0.001, 95% CI [–0.0635, ∞); L5, *z *= –0.507, *p* = 0.971, Cohen’s *d* = –0.0318, 95% CI [–0.0761, ∞). ***B***, We fit an LME model with stimulus category (naturalistic or artificial), area, and their interaction as fixed effects and found a significant interaction (likelihood ratio test, χ^2^(4) = 15.102, *p* = 0.00445). *Post hoc* one-sided *z*-tests (adjusted for multiple comparisons): V1, *z *= 0.612, *p* = 0.793, Cohen’s *d* = 0.076, 95% CI [–0.0483, ∞); LM, *z *= –0.136, *p* = 0.982, Cohen’s *d* = 0.0270, 95% CI [–0.0665, ∞); AL, *z *= 2.879, *p* = 0.00995, Cohen’s *d* = 0.226, 95% CI [0.0329, ∞); PM, *z *= –1.929, *p* > 0.999, Cohen’s *d* = –0.151, 95% CI [–0.161, ∞); AM, *z *= 2.318, *p* = 0.05005, Cohen’s *d* = 0.0984, 95% CI [–0.0179, ∞). ***A***, ***B***, Asterisks indicate significant *post hoc* one-sided *z*-tests in the layer (***A***) and area (***B***) interaction LME models (**p < 0.01, ***p < 0.001). Boxes indicate quartiles; whiskers indicate the minimum and maximum of data lying within 1.5 times the interquartile range of the 25% or 75% quartiles; diamonds indicate observations outside this range. ***C***, Mean values are indicated by bars. Download Figure 3-8, TIF file.

10.1523/ENEURO.0280-21.2021.f3-9 Extended Data Figure 3-9 Spectral differentiation analysis on ΔF/F_0_ traces with initial calcium transients removed. Transients were defined as the first 200 ms (6 imaging samples) of the signal after each L_0_ calcium event. These samples were replaced with linearly interpolated values and ND was calculated for the resulting signal. The difference in ND of responses to unscrambled versus scrambled stimuli is plotted for each session by layer (***A***), area (***B***), and layer-area pair (***C***). Results are similar to the main analysis shown in Figure 3, indicating that our ND results are not driven solely by initial transients in the calcium response. ***A***, We fit an LME model with stimulus category (naturalistic or artificial), layer, and their interaction as fixed effects and found a significant interaction (likelihood ratio test, χ^2^(2) = 6.024, *p* = 0.0492). *Post hoc* one-sided *z*-tests (adjusted for multiple comparisons): L2/3, *z *= 2.823, *p* = 0.00713, Cohen’s *d* = 0.175, 95% CI [0.0165, ∞); L4, *z *= 0.850, *p* = 0.483, Cohen’s *d* = 0.0568, 95% CI [–0.0299, ∞); L5, *z *= –0.674, *p* = 0.984, Cohen’s *d* = –0.0357, 95% CI [–0.0680, ∞). ***B***, We fit an LME model with stimulus category (naturalistic or artificial), area, and their interaction as fixed effects and found a significant interaction (likelihood ratio test, χ^2^(4) = 12.886, *p* = 0.0119). *Post hoc* one-sided *z*-tests (adjusted for multiple comparisons): V1, *z *= 1.032, *p* = 0.559, Cohen’s *d* = 0.0946, 95% CI [–0.0390, ∞); LM, *z *= –0.495, *p* = 0.997, Cohen’s *d* = –0.0354, 95% CI [–0.092, ∞); AL, *z *= 2.911, *p* = 0.00899, Cohen’s *d* = 0.257, 95% CI [0.0190, ∞); PM, *z *= –1.429, *p* > 0.999, Cohen’s *d* = –0.0966, 95% CI [–0.114, ∞); AM, *z *= 2.055, *p* = 0.0958, Cohen’s *d* = 0.125, 95% CI [–0.00800, ∞). ***A***, ***B***, Asterisks indicate significant *post hoc* one-sided *z*-tests in the layer (***A***) and area (***B***) interaction LME models (**p < 0.01). Boxes indicate quartiles; whiskers indicate the minimum and maximum of data lying within 1.5 times the interquartile range of the 25% or 75% quartiles; diamonds indicate observations outside this range. ***C***, Mean values are indicated by bars. Download Figure 3-9, TIF file.

First, we fit a LME model with cortical layer, stimulus category (unscrambled or scrambled), and their interaction as fixed effects, with experimental session as a random effect [lme4 formula: “differentiation ∼ 1 + layer * stimulus_category + (1 | session)”]. To test layer specificity, we then fit a reduced model with the interaction removed [“differentiation ∼ 1 + layer + stimulus_category + (1 | session)”] and used a likelihood ratio test to compare the two models.

Next, we fit an LME model with cortical area, stimulus category, and their interaction as fixed effects, with experimental session as a random effect [lme4 formula: “differentiation ∼ 1 + area * stimulus_category + (1 | session)”]. To test area specificity, we fit a reduced model with the interaction removed [“differentiation ∼ 1 + area + stimulus_category + (1 | session)”] and used a likelihood ratio test to compare the two models.

To test for differences in ND among the unscrambled continuous stimuli [“snake (predator),” “crickets (prey),” “man writing,” “mousecam,” and “conspecifics”; Fig. 7], we fit an LME model with stimulus as a fixed effect and experimental session as a random effect [lme4 formula: “differentiation ∼ 1 + stimulus + (1 | session)”] and used a likelihood ratio test to compare this to a reduced model without the stimulus term.

We visualized these results by plotting the difference in mean ND for each experimental session; however, no averaging was performed in the statistical analyses.

#### *Post hoc* tests

We performed *post hoc* one-sided *z*-tests to reject the null hypothesis that mean ND for scrambled ≥ mean ND for unscrambled in favor of the alternative hypothesis that mean ND for scrambled < mean ND for unscrambled using the glht function from the multcomp package in R on each LME model with contrasts between stimulus categories (unscrambled or scrambled) within each layer and area, respectively. *P* values were adjusted for multiple comparisons using the single-step method in multcomp (Hothorn et al., 2008).

*Post hoc* two-sided *z*-tests for pairwise differences among the unscrambled continuous stimuli were performed with the emmeans function from the emmeans package in R [“emmeans(model, pairwise ∼ stimulus)”, with *p* values adjusted for multiple comparisons using Tukey’s method; Lenth, 2020].

For all *post hoc* tests, simultaneous 95% confidence intervals (CIs) were obtained using the confint methods of the respective model objects. Effect sizes are reported as the Cohen’s *d* value for each pairwise comparison. Cohen’s *d* was calculated with the pooled SD:

$$\sqrt{\frac{\left(n_{1}-1\right)\sigma_{1}^{2}+(n_{2}-1)\sigma_{2}^{2}}{n_{1}+n_{2}-2}}.$$

#### Permutation tests

Permutation tests were performed for each experimental session to test whether spectral differentiation evoked by unscrambled stimuli was greater than that evoked by scrambled stimuli (Table 1). We obtained a null distribution by randomly permuting the trial labels (unscrambled or scrambled) 20,000 times and computing the difference in mean spectral differentiation on unscrambled and scrambled trials for each permutation. *P* values were computed as the fraction of permutations for which the permuted difference was greater than the observed difference, and significance is reported at the level of α = 0.05.

**Table 1:** Permutation tests show increased ND for unscrambled versus scrambled stimuli in L2/3 of AL and AM at the level of individual experimental sessions

	V1	LM	AL	PM	AM	All areas
L2/3	1/3	1/3	3/3	0/3	3/3	8/15
L4	0/3	1/3	0/3	0/3	0/3	1/15
L5	0/3	0/3	0/2	0/3	0/3	0/14
All layers	1/9	2/9	3/8	0/9	3/9	

Entries contain the fraction of sessions in which the mean ND of responses to unscrambled stimuli was significantly greater than responses to their scrambled counterparts at a threshold of α = 0.05. For each session, a null distribution was obtained by randomly permuting trial labels (unscrambled or scrambled) 20,000 times and computing the difference in mean ND on unscrambled and scrambled trials for each permutation. *P* values were computed as the fraction of permutations for which the permuted difference was greater than the observed difference.

#### Mediation analyses

Mediation analyses were conducted using the mediation package in R (Tingley et al., 2019).

We analyzed whether the mean event magnitude during a trial mediated the effect of stimulus category on differentiation values by fitting LME models for the mediator and outcome, including arousal variables as covariates, and using the mediate function [mediator model: “mean_magnitude ∼ 1 + stimulus_type + pupil_diameter + locomotion + (1 | session)”; outcome model: “differentiation ∼ 1 + mean_magnitude + stimulus_type + pupil_diameter + locomotion + (1 | session)”; treatment: “stimulus_category”; mediator: “mean_magnitude”]. This analysis assesses the contribution of the treatment variables on the outcome variable via each of two causal paths: (1) stimulus category and arousal level affect the mean event magnitude, which then in turn affects the measured ND (mediated); and (2) stimulus category and arousal directly affect the measured ND (direct).

For the analysis shown in Extended Data Figure 7-1, we fit LME models for each arousal variable (locomotion and pupil diameter) as a mediator, in each case including the other arousal variable as a covariate [e.g., “locomotion ∼ 1 + stimulus + pupil_diameter + (1 | session)”], and an outcome model [“differentiation ∼ 1 + stimulus + locomotion + pupil_diameter + (1 | session)”]. Mediation for a particular pair was evaluated using the mediate function with the “treat.value” and “control.value” arguments. For each stimulus pair identified as eliciting significantly different ND in our *post hoc* LME analyses, and for each arousal variable, this analysis assesses the contribution of the effect of stimulus on ND via each of two causal paths: (1) the stimulus affects arousal as measured by pupil diameter or locomotion, which then in turn affects ND (mediated); and (2) stimulus directly affects ND, independent of arousal level (direct).

### Decoding analyses

For each experimental session, we decoded stimulus category (unscrambled or scrambled) using linear discriminant analysis with the Python package scikit-learn (Pedregosa et al., 2011). First, the responses to each category were concatenated to form an *s* × (*n · t*) matrix, where *s* is the number of stimulus presentation trials, *n* is the number of cells recorded, and *t* is the number of two-photon imaging samples in a single trial. To obtain a tractable number of features for linear discriminant analysis, we used PCA to reduce the dimensionality of the matrix such that the number of components *c* was sufficient to retain 99% of the variance along the rows, yielding an *s *×* c* matrix [sklearn.decomposition.PCA(n_components = 0.99)]. This was then used to train a shrinkage-regularized LDA classifier with fivefold cross-validation [sklearn.discriminant_analysis.LinearDiscriminantAnalysis(solver='lsqr', shrinkage='auto')]. We report the mean balanced accuracy score (sklearn.metrics.balanced_accuracy_score) on the heldout test data across cross-validation folds. Chance performance is 0.5.

For Extended Data Figure 6-1, we used the same procedure as described above, but the classifier was trained to decode stimulus identity rather than category; chance performance is 1/12. For Extended Data Figure 7-2, we used the same procedure but trained the classifier using only responses to the five continuous naturalistic stimuli, and classifier performance was evaluated for each stimulus separately with the F1 score.

### Code accessibility

The analysis code used in this work is freely available online at https://github.com/wmayner/openscope-differentiation. The code is also available as Extended Data 1.

10.1523/ENEURO.0280-21.2021.ed1 Extended Data 1 Analysis code. All analyses and figures can be reproduced by following the instructions in README.md. Download Extended Data 1, ZIP file.

## Results

Using *in vivo* two-photon calcium imaging (Fig. 1*A–D*), we recorded from the left visual cortex of awake mice while they passively viewed stimuli presented to the contralateral eye. We used the transgenic mouse lines Cux2, Rorb, and Rbp4, in which GCaMP6f is expressed in excitatory neurons predominantly in L2/3, L4, and L5, respectively (three mice each; Cux2, two males; Rorb, three males; Rbp4, one male; see Materials and Methods, Transgenic mice). Visual cortical areas were delineated via ISI (Fig. 1*B*). Data were collected from L2/3, L4, and L5 in each of five areas (V1, LM, AL, PM, and AM; Fig. 1*E*) across 45 experimental sessions (15 sessions per transgenic line; 9 sessions per area; 5 ± 1 sessions per mouse; number of cells shown in Extended Data Fig. 1-2). Mice were head-fixed and free to move on a rotating disk while pupil diameter and running velocity were recorded. During each 70-min session, 12 30-s movie stimuli were presented in a randomized block design with 10 repetitions, with 4 s of mean-luminance gray shown between stimulus presentations (Fig. 1*F*,*G*; Extended Data Fig. 1-1). Stimuli were presented in greyscale but were not otherwise modified (in particular, it should be noted that spatial frequencies beyond the mouse acuity limit will appear blurred to the mice). Representative ΔF/F_0_ traces and behavioral data are shown in Figure 1*H*. One imaging session in L5 of AL was excluded from our analyses because of technical problems with the two-photon recording.

To measure ND, we employed a method from Mensen et al. (2018) for analyzing a set of timeseries recorded during the presentation of a continuous stimulus (Fig. 2). Briefly, the power spectrum of each cell’s ΔF/F_0_ trace was estimated in 1-s windows. The cells’ power spectra during simultaneous windows were concatenated to form a vector representing the neurophysiological state of the population during that window. We calculated ND for each trial as the median Euclidean distance between the 30 population states elicited over the course of the 30 s stimulus. We computed distances in the frequency domain rather than the time domain to focus on differences in overall population state rather than differences in precise timing of ΔF/F_0_ transients. To account for variability in the size of the imaged populations we divided ND values by the square root of the number of cells (see Materials and Methods, Spectral differentiation). Spectral differentiation is zero when the set of ΔF/F_0_ traces is perfectly periodic with a period of 1 s (the window size), and it is high when many traces exhibit temporally varied patterns across the 30 s (Extended Data Fig. 2-1). The measure scales with the magnitude of the signal and thus has no well-defined maximum.

To compare the differentiation of responses to naturalistic and artificial stimuli, we generated Fourier phase-scrambled versions of two of our movie stimuli. Phase-scrambling destroys the naturalistic structure of the stimulus while closely matching the power spectrum (the spectrum was not conserved exactly because of numerical representational limitations of the stimulus format; see Materials and Methods, Phase scrambling). Note that operations that leave the power spectrum of a signal unchanged will not affect its spectral differentiation.

For the “mouse montage 1” stimulus (a montage of six 5-s naturalistic movie clips), we performed the phase-scrambling in two ways: (1) along the temporal dimension, on each pixel independently; and (2) along the two spatial dimensions, on all pixels. For the “mousecam” stimulus (a continuous 30-s clip of movement at ground level through the underbrush of a forest) we performed only the spatial phase-scrambling. This yielded two unscrambled stimuli and three scrambled stimuli (Extended Data Fig. 1-1). The full set of twelve stimuli was designed to span different levels of putative ethological relevance; here, we focus on the comparison of the unscrambled stimuli to their scrambled versions because low-order stimulus statistics are controlled and thus the contrast can be more easily interpreted.

### Unscrambled stimuli elicit more differentiated responses compared with scrambled stimuli

We hypothesized that the unscrambled stimuli would elicit higher ND than their phase-scrambled counterparts. We tested this by fitting LME models with experimental session as a random effect (see Materials and Methods, LME models); mean differences in ND of responses to unscrambled versus scrambled stimuli are shown in Figure 3. We obtained similar results contrasting naturalistic versus artificial stimuli across the entire stimulus set (Extended Data Fig. 3-1). ND values were approximately log-normally distributed, so we applied a logarithmic transform to ND in all statistical analyses (see Materials and Methods, Statistical analyses).

#### Increased differentiation for unscrambled stimuli is specific to excitatory cells in L2/3

We found that unscrambled stimuli elicited more differentiated responses specifically in L2/3 (Fig. 3*A*). We fitted an LME model with stimulus category (unscrambled or scrambled), layer, and their interaction as fixed effects and found a significant interaction (likelihood ratio test, χ^2^(2) = 13.379, *p* = 0.00124). *Post hoc* tests showed that the unscrambled versus scrambled difference was specific to L2/3 [one-sided *z*-test; L2/3, *z *=* *3.866, *p* = 1.66e–4, Cohen’s *d *=* *0.164, 95% CI [0.051, ∞)^a^; L4, *z *=* *0.191, *p* = 0.810, Cohen’s *d *=* *0.011, 95% CI [–0.057, ∞)^b^; L5, *z *= –1.168, *p* = 0.998, Cohen’s *d* = –0.067, 95% CI [–0.100, ∞)^c^; *p* values and CIs adjusted for multiple comparisons].

#### Increased differentiation for unscrambled stimuli is specific to areas AL and AM

The increased ND in response to unscrambled stimuli was area-specific (Fig. 3*B*). We fitted an LME model with stimulus category, area, and their interaction as fixed effects and found a significant interaction (likelihood ratio test, χ^2^(4) = 15.203, *p* = 0.00430). *Post hoc* tests showed that the unscrambled versus scrambled difference was specific to AL and AM [one-sided *z*-test; V1, *z *=* *0.704, *p* = 0.748, Cohen’s *d *=* *0.054, 95% CI [–0.061, ∞)^d^; LM, *z *= –0.234, *p* = 0.989, Cohen’s *d* = –0.016, 95% CI [–0.097, ∞)^e^; AL, *z *=* *2.873, *p* = 0.0101, Cohen’s *d *=* *0.200, 95% CI [0.022, ∞)^f^; PM, *z *= –1.843, *p* > 0.999, Cohen’s *d* = –0.122, 95% CI [–0.157, ∞)^g^; AM, *z *=* *2.446, *p* = 0.0356, Cohen’s *d *=* *0.268, 95% CI [0.128, ∞)^h^; adjusted for multiple comparisons].

It is conceivable that these results are artifacts of our implementation of the spectral differentiation measure. To check the robustness of our findings, we performed a sensitivity analysis in which we systematically varied (1) the distance metric used to assess differences between population states; (2) the window length that defines the state of the neural population; (3) the frequency bin spacing in the spectra; and (4) the window function and amount of overlap used in the spectral estimation step (Extended Data Figs. 3-2, 3-3, 3-4). The results were qualitatively the same for nearly all combinations of these parameters we tested.

Since arousal state modulates neuronal activity in visual cortex (Niell and Stryker, 2010; Polack et al., 2013; Reimer et al., 2014; McGinley et al., 2015b; Vinck et al., 2015; Dadarlat and Stryker, 2017; Salkoff et al., 2020), the increase in firing rates seen during periods of high arousal raises the possibility that the differences in ND we observed could be due to changes in arousal alone rather than stimulus category. To rule this out, we repeated the sensitivity analysis of our main results while including locomotion and pupil diameter as covariates in the LME models. Consistent with the simpler models, for nearly all parameter combinations, L2/3 of AL and AM emerged as the cell populations in which ND is greater for unscrambled versus scrambled stimuli (Extended Data Figs. 3-5, 3-6, 3-7), indicating that the measured arousal variables are insufficient to fully explain the differences in ND we observed. We also analyzed whether the mean magnitude of calcium events (a proxy for firing rate) mediated the effect of stimulus category and found evidence for both mediated and direct effects (mediated effect: 0.1351, 95% CI [0.0784, 0.20]^i^, *p* < 2e–16; direct effect: 0.0951, 95% CI [0.0400, 0.15]^j^, *p* = 0.002; proportion of total effect mediated: 0.5898, 95% CI [0.3963, 0.80]^k^, *p* < 2e–16). That is, unscrambled stimuli led to increased ND relative to scrambled stimuli both directly, independent of mean event magnitude, and indirectly, via increases in mean event magnitude that in turn increased ND. Thus, while a portion of the effect of stimulus category was mediated by changes in population firing rate, this mediated effect is likewise insufficient to fully explain our results.

### Permutation tests for individual experimental sessions

The above analysis shows that the mean ND elicited by unscrambled stimuli is greater than that elicited by their phase-scrambled counterparts, and that this effect is driven by L2/3 cells in areas AL and AM. We also analyzed ND at the level of individual sessions with nonparametric permutation tests. For each session, we obtained a null distribution by randomly permuting the trial labels (unscrambled or scrambled) 20,000 times and computing the difference in mean ND on unscrambled versus scrambled trials for each permutation. *P* values were computed as the fraction of permutations for which the permuted difference was greater than the observed difference.

The results of the individual session analyses were consistent with the LME analyses (Table 1). In all sessions recorded from L2/3 of AL and AM, responses to unscrambled stimuli were significantly more differentiated than to scrambled stimuli (*p* < 0.05).

### Arousal is correlated with effect size

Locomotion and pupil diameter can be considered behavioral indications of engagement with the environment (Bennett et al., 2013; Ganea et al., 2020; Jacobs et al., 2020). We found that in L2/3 of AL and AM, effect sizes were positively correlated with locomotion activity (Pearson’s *r *=* *0.896; two-sided *t* test; *t*_(4)_ = 4.030, *p* = 0.0157, 95% CI [0.308, 1.00]^l^; Fig. 4, top left) and pupil diameter (*r *=* *0.716; *t*_(4)_ = 2.054, *p* = 0.109, 95% CI [–0.227, 1.00]^m^; Fig. 4, top right), suggesting that the difference in ND is more clear when the animal is engaged. However, we note that the relatively restricted range of observed mean locomotion fraction and pupil diameter values in the sessions of interest limits the generalizability of these conclusions.

### Multivariate analysis also shows increased differentiation for unscrambled stimuli

Spectral differentiation is a univariate measure in the sense that the coordinates of the population state vectors are orthogonal, so that each squared difference term in the Euclidean distance reflects differences only within a given cell’s responses across time. To ensure that our results were not due to this method of measuring ND, we also employed a multivariate approach that considers spatiotemporal differences in activity patterns across the cell population. For each session, the dimensionality of the population response vectors was reduced to 8 using UMAP (McInnes et al., 2020). In the resulting 8-dimensional space, ND was measured as the mean Euclidean distance to the centroid of the set of responses corresponding to that stimulus (see Materials and Methods, Multivariate differentiation).

The results of the multivariate analysis were consistent with those found using the spectral differentiation measure. The mean centroid distance was higher in response to unscrambled compared to scrambled stimuli (Fig. 5), and this effect was specific to L2/3 [layer × stimulus category interaction: likelihood ratio test, χ^2^(2) = 18.135, *p* = 1.154e–4; *post hoc* one-sided *z*-tests: L2/3, *z *=* *5.149, *p* = 3.92e–7, Cohen’s *d *=* *0.194, 95% CI [0.0181, ∞)^n^; L4, *z *=* *1.749, *p* = 0.116, Cohen’s *d *=* *0.0994, 95% CI [–0.00221, ∞)^o^; L5, *z *= –0.938, *p* = 0.995, Cohen’s *d* = –0.0651, 95% CI [–0.0189, ∞)^p^] and areas AL and AM [area × stimulus category interaction: likelihood ratio test, χ^2^(4) = 16.232, *p* = 0.00272; *post hoc* tests: V1, *z *=* *0.420, *p* = 0.872, Cohen’s *d *=* *0.0281, 95% CI [–0.0146, ∞)^q^; LM, *z *= –0.047, *p* = 0.974, Cohen’s *d* = –0.00269, 95% CI [–0.0182, ∞)^r^; AL, *z *=* *2.941, *p* = 0.00816, Cohen’s *d *=* *0.184, 95% CI [0.00508, ∞)^s^; PM, *z *=* *0.152, *p* = 0.945, Cohen’s *d *=* *0.0119, 95% CI [–0.0167, ∞)^t^; AM, *z *=* *4.436, *p* = 2.29e–5, Cohen’s *d *=* *0.277, 95% CI [0.0163, ∞)^u^].

10.1523/ENEURO.0280-21.2021.f5-1 Extended Data Figure 5-1 Multivariate differentiation analysis of detected calcium events. The mean difference in the mean centroid distance of detected-event responses to unscrambled versus scrambled stimuli is plotted for each session by layer (***A***), area (***B***), and layer-area pair (***C***). Multivariate differentiation elicited by unscrambled versus scrambled stimuli is higher in L2/3 of AL and AM, as well as the additional finding of higher differentiation in L2/3 of V1. ***A***, ***B***, Asterisks indicate significant *post hoc* one-sided *z*-tests in the layer (***A***) and area (***B***) interaction LME models (*p < 0.05, **p < 0.01, ***p < 0.001). Boxes indicate quartiles; whiskers indicate the minimum and maximum of data lying within 1.5 times the interquartile range of the 25% or 75% quartiles; diamonds indicate observations outside this range. ***C***, Mean values are indicated by bars. Download Figure 5-1, TIF file.

The multivariate differentiation measure is also suitable for use on discrete data. To support our main findings, we analyzed discrete calcium events detected from the ΔF/F_0_ traces with an L_0_-regularized algorithm (see Materials and Methods, Event detection). The results of multivariate differentiation analysis of these data were consistent with our results using ΔF/F_0_ traces (Extended Data Fig. 5-1), with the additional finding of significantly greater differentiation for unscrambled versus scrambled stimuli in L2/3 of V1 as well as AL and AM [layer × stimulus category interaction: likelihood ratio test, χ^2^(2) = 15.029, *p* = 0.000545; *post hoc* one-sided *z*-tests: L2/3, *z *= 5.337, *p* = 1.42e–7, Cohen’s *d *=* *0.178, 95% CI [0.0158, ∞)^v^; L4, *z *= 1.698, *p* = 0.128, Cohen’s *d *= 0.0611, 95% CI [–0.00208, ∞)^w^; L5, *z *= –0.124, *p* = 0.909, Cohen’s *d* = –0.00377, 95% CI [–0.0114, ∞)^x^; area × stimulus category interaction: likelihood ratio test, χ^2^(4) = 20.854, *p* = 0.000339; *post hoc* tests: V1, *z *= 3.132, *p* = 0.00434, Cohen’s *d *=* *0.167, 95% CI [0.00516, ∞)^y^; LM, *z *= –0.798, *p* > 0.999, Cohen’s *d* = –0.0451, 95% CI [–0.0198, ∞)^z^; AL, *z *= 2.757, *p* = 0.0145, Cohen’s *d *=* *0.100, 95% CI [0.00295, ∞)^aa^; PM, *z *= –0.366, *p* = 0.994, Cohen’s *d* = –0.0160, 95% CI [–0.0170, ∞)^bb^; AM, *z *= 4.372, *p* = 3.07e–5, Cohen’s *d *=* *0.158, 95% CI [0.0130, ∞)^cc^].

### Decoding analysis does not reveal layer or area specificity

We next asked whether the layer and area specificity of our ND results would be reflected in our ability to decode the stimulus category (unscrambled or scrambled) from population responses. We performed fivefold cross-validated linear discriminant analysis to decode stimulus category for each session and scored the classifier using balanced accuracy (see Materials and Methods, Decoding analyses). Decoding performance was high for most areas and layers (Fig. 6), in contrast to the unscrambled-scrambled difference in ND. Performance was also high across layers and areas when we decoded stimulus identity, rather than category, using responses to all 12 stimuli (Extended Data Fig. 6-1).

10.1523/ENEURO.0280-21.2021.f6-1 Extended Data Figure 6-1 Stimulus identity can be accurately decoded from most layers and areas using responses to all 12 stimuli. Each point represents the mean fivefold cross-validated balanced accuracy score of linear discriminant analysis performed on a single session (see Materials and Methods, Decoding analyses). Chance performance is 1/12, indicated by the dotted line. Download Figure 6-1, TIF file.

### Differences in ND among individual stimuli

We also investigated whether ND differed among stimuli within the same category. This analysis was restricted to the set of unscrambled stimuli without jump cuts, i.e., the five naturalistic continuous 30-s clips, to avoid potential confounds in comparing stimuli with and without abrupt transitions between different scenes. Here, we used data from all layers and areas, since although responses from L2/3 of AL and AM drove the unscrambled/scrambled differences, within-category differences might not be restricted to that subset. We fitted an LME model with stimulus as a fixed effect and found it was significant (likelihood ratio test, χ^2^(4) = 32.115, *p* = 1.812e–6). *Post hoc* pairwise two-sided *z*-tests (adjusted for multiple comparisons), shown in Figure 7, revealed that the predator stimulus (a snake) evoked significantly higher differentiation than clips of conspecifics (*z *=* *3.229, *p* = 0.0110, Cohen’s *d *=* *0.156, 95% CI [0.015, 0.180]^dd^), prey (crickets; *z *= 3.928, *p* = 8.149e–4, Cohen’s *d *=* *0.181, 95% CI [0.036, 0.201]^ee^), and a man writing (*z *=* *5.249, *p* = 1.522e–6, Cohen’s *d *=* *0.232, 95% CI [0.076, 0.241]^ff^). The “mousecam” clip of movement through a wooded environment also evoked significantly higher differentiation than the clip of a man writing (*z *=* *3.396, *p* = 0.00615, Cohen’s *d *=* *0.154, 95% CI [0.020, 0.185]^gg^). Mediation analysis showed a mixture of direct and arousal-mediated effects, indicating that changes in arousal cannot fully account for these differences (Extended Data Fig. 7-1). Here, we present the main effect of stimulus; for an exploration of interactions with layer and area, and a comparison to decoding, see Extended Data Figure 7-2.

10.1523/ENEURO.0280-21.2021.f7-1 Extended Data Figure 7-1 Mediation analysis of the effect of stimulus. To disentangle the effects of stimulus and arousal on ND, we performed a causal mediation analysis (see Materials and Methods, Mediation analyses). For each of the four stimulus pairs identified as having significantly different levels of ND in our *post hoc* analysis, we asked whether each of the arousal variables (locomotion or pupil diameter) was a mediator of the effect of stimulus on differentiation, in each case including the other arousal variable as a covariate. The analysis revealed a mixture of direct and mediated effects. Notably, for the largest contrast (predator vs man writing), for both locomotion and pupil diameter we found evidence only for a direct effect. Overall, we conclude that arousal alone cannot account for differences in ND among continuous, naturalistic stimuli. Download Figure 7-1, TIF file.

10.1523/ENEURO.0280-21.2021.f7-2 Extended Data Figure 7-2 Within-category differences in ND versus within-category differences in decoding performance, by layer and area. Top, Cohen’s *d* for pairwise mean differences in ND among naturalistic stimuli without jump cuts. Bottom, Cohen’s *d* for pairwise mean differences in stimulus identity decoding performance. For each session, we trained a linear discriminant analysis classifier using only responses to these five stimuli; classification performance was evaluated as the mean fivefold cross-validated F1 score for each stimulus (see Materials and Methods, Decoding analyses). Download Figure 7-2, TIF file.

### SD does not explain ND

It is possible that ND does not reflect functionally relevant visual processing but is instead merely inherited from the differentiation of the stimulus itself. To rule out this possibility, we computed the SD by treating each pixel of the stimulus as a “cell” and applying the spectral differentiation measure to the traces of pixel intensities over time after blurring the stimulus to account for the coarseness of mouse vision (see Materials and Methods, Spectral differentiation). Within L2/3 of AL and AM, the mean ND elicited by each stimulus was positively correlated with SD (Pearson’s *r *=* *0.746, one-sided *t* test; *t*_(10)_ = 3.542, *p* = 0.00267, 95% CI [0.393, 1.00]^hh^; Fig. 8). However, the noise stimulus is a highly influential observation (Cook’s *D *=* *2.318, an order of magnitude larger than the next most influential observation). If we exclude this stimulus, we find a weaker correlation (*r = *0.258; one-sided *t* test; *t*_(9)_ = 0.801, *p* = 0.222, 95% CI [–0.307, 1.00]^ii^). Furthermore, there was no evidence of a relationship with ND when considering only the scrambled stimuli and their unscrambled counterparts (*r* = –0.378; two-sided *t* test; *t*_(3)_ = –0.708*, p* *=* 0.530, 95% CI [–0.945, 0.756]^jj^). Thus, we conclude that ND is not inherited from SD. We also did not find a relationship with stimulus luminance, contrast, or spectral energy (Extended Data Fig. 8-1).

10.1523/ENEURO.0280-21.2021.f8-1 Extended Data Figure 8-1 ND versus low-level stimulus characteristics. ND is plotted against the mean luminance, contrast, and spectral energy of the stimuli. Mean luminance was computed as the average pixel intensity. Contrast was calculated as the SD of pixel intensities. Spectral energy of the blurred stimuli was computed as the sum of the energy spectral density of each pixel’s intensity timeseries after removing the DC component. Download Figure 8-1, TIF file.

## Discussion

Our results show that excitatory L2/3 neurons in visual areas AL and AM have more differentiated responses to stimuli with naturalistic structure than to phase-scrambled stimuli with closely matched low-order statistics, indicating that these populations are uniquely sensitive to high-level natural features in this stimulus set. We found this difference at the level of single experimental sessions, and it was robust to complementary methods of measuring ND. Effect sizes were larger with increasing pupil diameter and locomotion, suggesting sensitivity to the animal’s arousal level. Decoding analysis showed a marked lack of area and layer specificity: stimulus category could be accurately decoded from the activity of most cell populations we surveyed. In addition to the differences between unscrambled and scrambled stimuli, we found differences in ND among unscrambled stimuli. Finally, we argued that ND is not merely inherited from the differentiation of the stimulus.

The precise functional specialization of visual areas in the mouse remains unclear (Glickfeld and Olsen, 2017). Recent large-scale anatomical (Harris et al., 2019) and functional (Siegle et al., 2021b) studies have uncovered a “shallow hierarchy” in which V1 lies at the base, followed by LM, RL, AL, and PM, with AM at the top. In this light, our finding that ND in L2/3 of AL and AM is sensitive to high-level naturalistic structure could be interpreted as a reflection of hierarchical processing, which may be constructing a richer dynamical repertoire for perception of naturalistic stimuli at higher hierarchical levels. Interestingly, we did not find this effect in PM, despite its intermediate position between AL and AM in the hierarchy, suggesting that such hypothetical processing toward richer repertoires is not fully determined by the one-dimensional hierarchy, but may involve specific pathways through subsets of visual areas. These observations indicate that differentiation analysis may help refine our understanding of functional specialization of brain areas and uncover differences between them that can be used to direct further investigations.

A recent study found that feedback projections from higher visual areas to L2/3 excitatory neurons in V1 create a second RF surrounding the feedforward RF and that these RFs are mutually antagonistic, pointing to a role for these neurons in predictive processing (Keller et al., 2020). If this pattern is present at higher levels of the visual hierarchy, then the layer specificity we find could be explained by a scenario in which feedback to AL and AM from areas higher in the putative dorsal stream (Marshel et al., 2011; Wang et al., 2012) are integrated with feedforward inputs in L2/3 to compute prediction errors about high-level visual features. In this scenario, the naturalistic stimuli, which contain high-level features that are presumably less predictable, would elicit more prediction errors and thus more differentiated activity.

Stimulus-evoked activity in cortex is modulated by arousal level and behavioral state (McGinley et al., 2015b; Salkoff et al., 2020). Locomotion is associated with heightened arousal, increased membrane depolarization, firing rates, and signal-to-noise ratio, and enhanced stimulus encoding (Niell and Stryker, 2010; Bennett et al., 2013; Polack et al., 2013; Vinck et al., 2015; Dadarlat and Stryker, 2017). Pupil diameter can serve as an index of arousal (McGinley et al., 2015a,b; Larsen and Waters, 2018). Larger pupil size is associated with increases in the gain, amplitude, signal-to-noise ratio, and reliability of responses in V1 (Reimer et al., 2014). Thus, our finding that increased pupil diameter and locomotion are associated with larger effect sizes could be explained by an increase in response gain or amplitude in V1 that is inherited by downstream AL and AM: since the ND in these areas is selective for naturalistic structure, increased bottom-up drive could accentuate unscrambled-scrambled differences in ND.

Alternatively, response gain or amplitude in higher visual areas could be modulated directly by subcortical arousal systems. The noradrenergic and cholinergic systems are likely candidates, although it is not clear why noradrenergic modulation would cause an effect specific to L2/3; as for cholinergic modulation, Pafundo et al. (2016) showed that V1 and LM are differentially modulated by basal forebrain stimulation such that the response gain and reliability of excitatory L2/3 neurons was enhanced in V1 but not in LM, despite an even distribution of basal forebrain axons across all layers in both areas. However, neuromodulatory regulation of activity in other visual areas, in particular AL and AM, has not yet been characterized in great detail. Another possibility is a top-down effect, where increases in arousal and locomotion reflect increased attentional engagement that favors processing of high-level stimulus features, selectively increasing ND for the unscrambled stimuli. In the passive viewing paradigm employed here, in which the animal is not motivated to attend to the stimuli, the top-down modulation of sensory processing may vary considerably across the experimental session as arousal and attention fluctuate.

Although differentiation analysis revealed area-specific and layer-specific differences in responses to unscrambled and phase-scrambled stimuli, our ability to decode stimulus category from neural responses was remarkably similar across areas and layers. These findings are consistent with a growing literature that reveals a dissociation between encoding and function (Erlich et al., 2015; Katz et al., 2016; Tsunada et al., 2016; Liu and Pack, 2017; Jin and Glickfeld, 2020; Zatka-Haas et al., 2021). The contrast between our ND and decoding results highlights an important distinction: decoding reveals information content, but this information is necessarily measured from the extrinsic perspective (Tononi, 2004; Oizumi et al., 2014; Tononi et al., 2016; Buzsáki, 2019). The presence of information about a stimulus in a neural circuit does not imply that the information is functionally relevant (Brette, 2019). As an extreme example, stimulus category would presumably be perfectly decodable from photons impinging on the retina, but this would reveal nothing of interest about perception. By contrast, ND is an intrinsic measure defined without reference to a stimulus (Boly et al., 2015; Mensen et al., 2017, 2018). In the brain, a complex evolved system in which activity is energetically costly, ND may be a signature of functionally relevant dynamics. The dissociation we find between ND and decoding indicates that differentiation analysis can point to populations of interest that are not revealed by detecting stimulus information.

Finally, we also found that the predator stimulus and the “mousecam” stimulus elicited significantly higher ND than other unscrambled continuous stimuli. The predator stimulus finding is intriguing because that stimulus has lower luminance, contrast, and spectral energy than the clip of conspecifics in a home cage (Extended Data Fig. 8-1); given the importance of detecting natural predators, the high ND evoked by this stimulus may reflect its salience to the visual system, driven by high-level features such as the presence of the predator rather than low-order stimulus statistics. This also demonstrates that differentiation analysis can probe differences in visual responses at the level of individual stimuli.

It is important to note the limitations of these data. First, calcium imaging provides an imperfect proxy for neuronal activity. The fluorescence signal from calcium indicators is more sensitive to bursts of spikes than sparse, low-frequency spiking (Chen et al., 2013; Pachitariu et al., 2018; Ledochowitsch et al., 2019; Wei et al., 2020; Huang et al., 2021; Siegle et al., 2021a). Such sparse activity may contribute to ND but would not be present in this dataset. However, given the typically sparse spiking activity of L2/3 excitatory neurons compared with deeper layers (Barth and Poulet, 2012), it is possible that this limitation only obscures even stronger L2/3 specificity. Second, for this exploratory study we used a range of naturalistic stimuli and a limited number of phase-scrambled control stimuli to include diverse high-level features. Future studies could test our findings using a larger set of artificial stimuli controlling for other low-level characteristics, e.g., optical flow, in addition to the power spectrum. Third, the restricted range of the average arousal measures we observed in our experiments in L2/3 of AL and AM limits the generalizability of the association we observed between effect size and arousal state. Fourth, while we observed medium to very large effect sizes within individual experimental sessions in L2/3 of AL and AM (Cohen’s *d *=* *0.57–1.25), the overall effect was relatively subtle (Cohen’s *d *=* *0.34) because of variability in ND values across sessions. There was also considerable variability in arousal state and locomotor activity across trials. To the extent that these factors modulate effect size, future work might uncover larger effects by employing an active paradigm where the animal is motivated to attend to the stimuli.

In summary, we measured stimulus-evoked differentiation of neural activity with cellular resolution and found increased ND in response to unscrambled versus scrambled stimuli. This effect was specific to L2/3 excitatory cells in AL and AM and was enhanced at higher arousal levels. To our knowledge, this study is the first to systematically measure stimulus-evoked differentiation with cellular resolution across multiple cortical areas and layers. These results advance our understanding of the functional differences among visual areas, and future work should integrate our findings into the emerging picture of a shallow hierarchy in the mouse visual system, for example by investigating potential differences in neuromodulation among areas or the contrast between AL/AM and PM. Differentiation analysis is motivated by IIT, and provides an intrinsic, “inside-out” analytical approach that complements extrinsic, “outside-in” measures such as decoding performance, which in this dataset did not distinguish specific cell populations. This method can be used to compare individual stimuli and may provide a readout of the degree to which a given stimulus induces a rich and varied perceptual experience. Future studies should investigate stimulus-evoked differentiation with cellular resolution in humans and nonhuman primates, where subjective reports are available, and thereby determine the contributions of distinct cell populations to ND while correlating ND with phenomenology.

**Table 2:** Statistics

	Data structure	Type of test	95% CI
a	Normal	*z* (one-sided)	[0.051, ∞)
b	Normal	*z* (one-sided)	[–0.057, ∞)
c	Normal	*z* (one-sided)	[–0.100, ∞)
d	Normal	*z* (one-sided)	[–0.061, ∞)
e	Normal	*z* (one-sided)	[–0.097, ∞)
f	Normal	*z* (one-sided)	[0.022, ∞)
g	Normal	*z* (one-sided)	[–0.157, ∞)
h	Normal	z (one-sided)	[0.128, ∞)
i	Normal	Imai et al. (2010)	[0.0784, 0.20]
j	Normal	Imai et al. (2010)	[0.0400, 0.15]
k	Normal	Imai et al. (2010)	[0.3963, 0.80]
l	Normal	*t* (two-sided)	[0.308, 1.00]
m	Normal	*t* (two-sided)	[–0.227, 1.00]
n	Normal	*z* (one-sided)	[0.0180, ∞)
o	Normal	*z* (one-sided)	[–0.00221, ∞)
p	Normal	*z* (one-sided)	[–0.0189, ∞)
q	Normal	*z* (one-sided)	[–0.0146, ∞)
r	Normal	*z* (one-sided)	[–0.0182, ∞)
s	Normal	*z* (one-sided)	[0.00507, ∞)
t	Normal	*z* (one-sided)	[–0.0167, ∞)
u	Normal	*z* (one-sided)	[0.0163, ∞)
v	Normal	*z* (one-sided)	[0.0158, ∞)
w	Normal	*z* (one-sided)	[–0.00208, ∞)
x	Normal	*z* (one-sided)	[–0.0114, ∞)
y	Normal	*z* (one-sided)	[0.00516, ∞)
z	Normal	*z* (one-sided)	[–0.0198, ∞)
aa	Normal	*z* (one-sided)	[0.00295, ∞)
bb	Normal	*z* (one-sided)	[–0.0170, ∞)
cc	Normal	*z* (one-sided)	[0.0130, ∞)
dd	Normal	*t* (two-sided)	[0.015, 0.180]
ee	Normal	*t* (two-sided)	[0.036, 0.201]
ff	Normal	*t* (two-sided)	[0.076, 0.241]
gg	Normal	*t* (two-sided)	[0.020, 0.185]
hh	Normal	*t* (one-sided)	[0.393, 1.00]
ii	Normal	*t* (one-sided)	[–0.307, 1.00]
jj	Normal	*t* (two-sided)	[–0.945, 0.756]

## Synthesis

Reviewing Editor: Arianna Maffei, Stony Brook University Decisions are customarily a result of the Reviewing Editor and the peer reviewers coming together and discussing their recommendations until a consensus is reached. When revisions are invited, a fact-based synthesis statement explaining their decision and outlining what is needed to prepare a revision will be listed below. The following reviewer(s) agreed to reveal their identity: NONE.

The reviewers agreed on the significance of the results and approach. They raised a number of concerns regarding the interpretation of the data and on some aspects of the methodology presented in the manuscript.

The main points of concern regard 1) the features of neuronal activity that are captured by ND (and the degree to which this might be biased by indicator kinetics) and 2) the potentially complicated relationship between firing rate, arousal and salient stimuli.

The uneven distributions of pupil diameter and locomotion, which may make the interpretation of the arousal data difficult. Both reviewers agree that this point should be at least addressed in the discussion.

The full set of reviewers comments is provided below.

Reviewer #1

The authors use a set of transgenic mice with layer-specific GCaMP expression and measure ND in response to a variety of naturalistic and artificial stimuli in V1 and 4 higher visual areas. Their main finding is that measures of ND in populations of L2/3 neurons in AL and AM (but not in other areas/layers) can differentiate naturalistic from scrambled movies. This result is robust to a range of analysis parameters, is enhanced by arousal, and provides specificity that is not present using classical decoding approaches. In addition, this measure differentiates activity in response to a movie with a predator compared to other naturalistic movies, although this effect is consistent across most areas/layers and doesn’t diverge from classical decoding results. Overall, this is a creative approach to investigating coding in the visual system that appears to provide some functional insights, and will be of interest to groups investigating how circuits encode sensory information across species and systems. I only have some small concerns and questions for clarification.

1. In the introduction, the authors take a very forceful position on the importance and potential of ND:

Line 54- “ND must underlie phenomenological differentiation"

Line 65- “...which neuronal populations contribute directly to generating conscious percepts... Differentiation analysis can shed light on this question...”

While ND does seem like a promising unbiased approach for studying the complexity of neuronal signals, the degree to which it may or may not be the substrate for conscious perception is still up for debate, and there certainly does not need to be a causal (or tautological) relationship. The authors do not need to oversell the importance of this measure and should take a slightly more circumspect approach in the introduction.

2. The measure of ND investigates neuronal activity in the frequency domain. The authors do a good job of explaining how the calcium signals that they measure may not reflect the true dynamics of neuronal firing. However, it is nonetheless surprising that the richness of ND is revealed in the 1s windows used for the Fourier transforms given slow decay tau of the indicator. It is even more surprising that the results are essentially unchanged when the window is only 200 ms since this is only 6 imaging samples. This suggests that ND might be largely identifying the initial transients in the neuronal activity rather than more complex dynamics. The authors should discuss (or analyze) what features of the neuronal activity ND using to distinguish responses to stimuli. It would also be worthwhile to see whether differences in the kinetics of responses differ across layers/areas and whether this could account for any of the differences in ND.

3. Additional clarification is needed on how the data were analyzed and combined.

a. Is the Fourier transform performed on single trial responses, or on trial (movie) averaged data?

b. Are the single data points (for instance in figure 3) the average of the different combinations of three pairs of natural/scrambled movies or just one pair? Similarly, is the LME analysis done with these separated or averaged?

c. How was the standard deviation calculated that is used to derive Cohen’s d?

4. The correlation of ND with arousal is interesting. Can this magnitude of effect be explained by the increases in firing rate that are expected during arousal? The authors should run some simulations to determine whether this is sufficient to explain the result.

5. The control analyses of the stimulus differentiation are important. However, the approach of treating stimulus pixels independently seems inappropriate given that we know the resolution of mouse vision is much coarser. This analysis should be repeated using a gaussian blur the size of a receptive field in mouse V1.

6. The effect size is generally less than 1 std deviation- the authors should discuss whether this is a large or subtle effect.

7. The use of three repeats per layer per area seems like the minimum, especially given the degree of variability shown in figure 3c. The authors should explicitly justify the use of this minimal dataset, and discuss the likelihood of identifying effects in other areas/layers with a larger dataset.

8. Line 375 states that there are 5 sessions per area- shouldn’t this be 9 (three repeats of three layers)?

Reviewer #2

The manuscript aims to provides a novel characterization of how a measure of neural differentiation may enable to differentiate the activity of visual cortical areas when meaningful stimuli compared to non-informative stimuli with similar features are shown to mice. This “inside-out approach” would contrast with the “outside-in” perspective provided by decoding (which in contrast allows to similarly decode meaningful as well as scrambled stimuli).

The analyses are in general carefully done. Nevertheless, I am concerned about the interpretation of the neural differentiation measure, specifically in relation to the analyses shown in Fig. 4 and 7.

1) Relationship between ND and arousal

Fig. 4 shows that there’s a positive relationship between ND and locomotion/pupil size for L2/3 neurons in AL and AM. I find that these results might simply be the consequence of a selection bias. In the top panel, the authors perform the correlation on a selection of points taken from a relatively small range for pupil size and especially for locomotion. This contrasts with the bottom panel, in which the analysis is done on much larger ranges and taking all points together. One cannot therefore exclude that: a) other layers/areas might show a similar correlation (see e.g. L4 in LM), and b) that the positive correlation found in L2/3 or AL and AM would not be present had measurement be done across the full range of pupil size and locomotion values. The authors should therefore: a) repeat the correlation analysis separately for each layer/area; b) if measurements for specific combinations of layers and areas show restricted ranges of pupil size and locomotion, attempt to expand the dataset or at least indicate this explicitly in the results section and in the discussion

2) Relationship between ND, stimulus content (and arousal)

Interestingly, Fig. 7 shows that ND values are higher for stimuli with a behavioral relevance (e.g. predators) compared to neutral stimuli. The authors suggest that this might be due to the salience of such stimuli. Indeed, in my opinion this may strongly relate to the positive relationship between arousal and ND (Fig. 4). If this positive relationship holds (see my point above), this may just indicate that ND does not reflect stimulus processing, but rather just arousal. Indeed, arousal is known to vary in mice on the time scale of seconds (or even faster) and reflect ongoing sensory processing. The authors need to verify whether specific sensory stimuli (e.g. prey) lead to a higher level of arousal and disentangle if the increased ND associated with certain stimuli might just be a consequence of such heightened arousal.

3) Interpretation of the results

The authors claim that ND “may provide a readout of the degree to which a given stimulus induces a rich and varied perceptual experience”. In view of my previous comments, I think that the authors need to first verify whether a simpler explanation might be possible, i.e. if arousal (here meant in terms of both pupil size and locomotion, although the two have been shown to differently impact visual processing) might simply explain the different ND levels reported by the authors. If this were the case, I believe that the manuscript would still report valuable results, but the interpretation of these would of course need to be significantly modified.

4) Limitations due to 2photon imaging

As the authors correctly state, 2-photon calcium imaging does not fully reflect spiking activity. Nevertheless, it would be valuable, in my opinion, to verify that the presented results still hold when applied to an estimate of spiking activity. For instance, could the author compute multivariate differentiation not on the raw dF/F traces, but on a deconvolved spiking trace? This analysis, when done on a set of recording sessions and compared to the already done measurements, would provide further support to the authors’ claim.
