# Supplementary figures and images for: Measuring Stimulus-Evoked Neurophysiological Differentiation in Distinct Populations of Neurons in Mouse Visual Cortex
Source: eNeuro. 2022 Feb 8;9(1):ENEURO.0280-21.2021. doi: 10.1523/ENEURO.0280-21.2021 (PMC8856714; doi:10.1523/ENEURO.0280-21.2021)

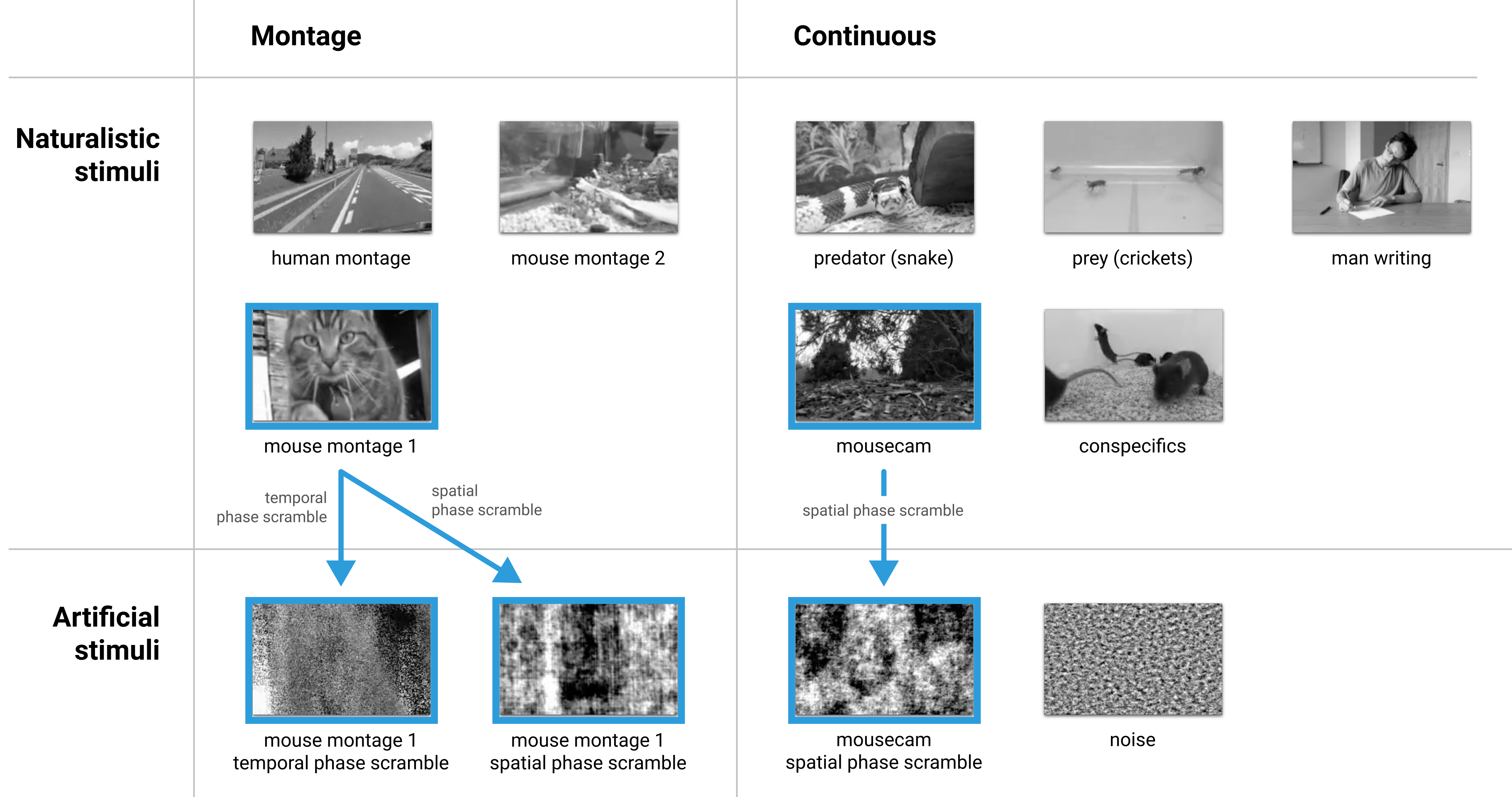

Supplement: Extended Data Figure 1-1 — Stimuli. Twelve 30-s-long greyscale naturalistic (top) and artificial (bottom) movie stimuli were presented. Left, Montages of six 5-s clips. Right, Continuous 30-s clips. Stimuli used in the main analysis are outlined in blue. Arrows indicate the phase-scrambling procedures. Download Figure 1-1, TIF file. [file enu-eN-NWR-0280-21-s03.tif]

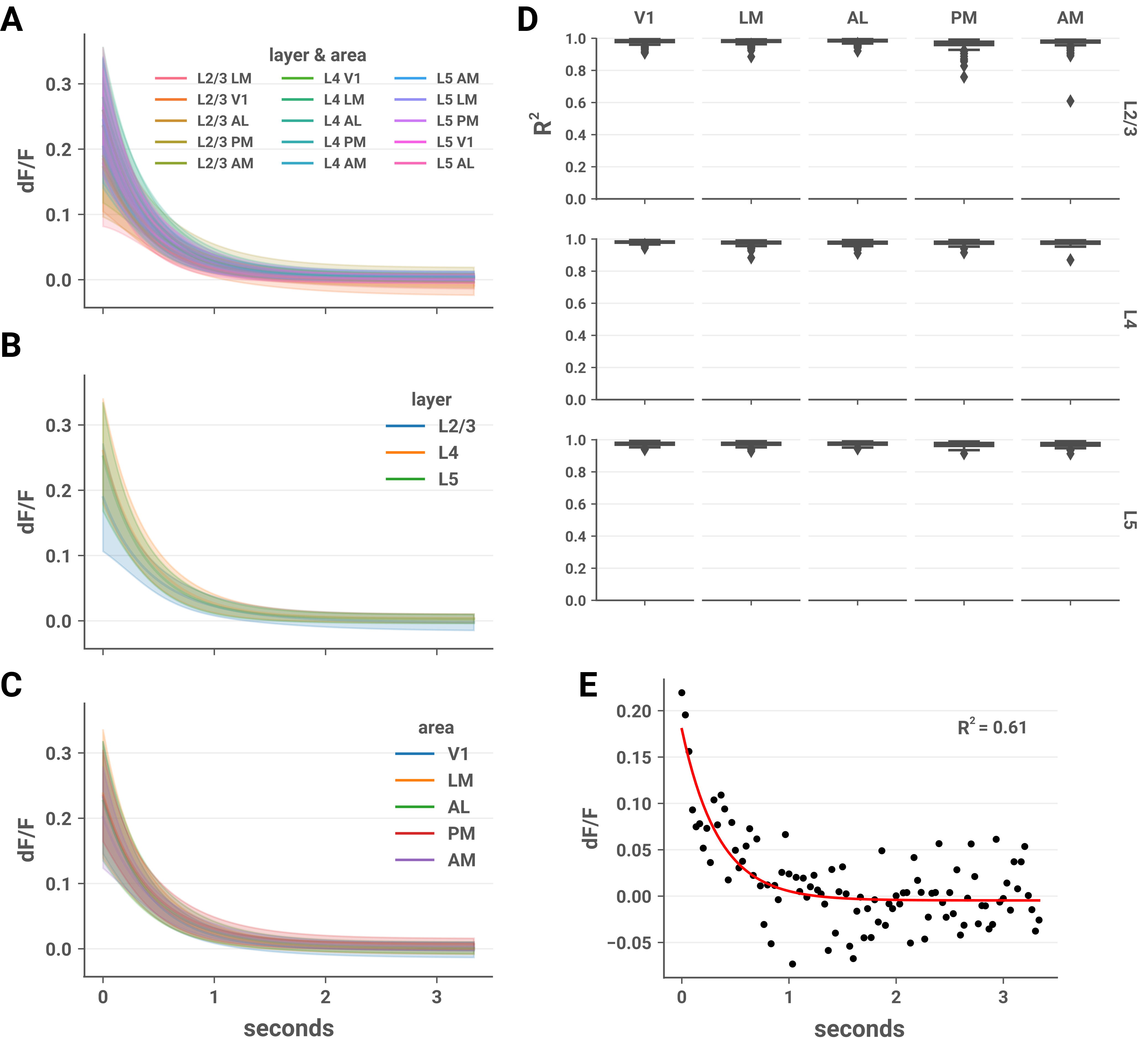

Supplement: Extended Data Figure 1-3 — Calcium indicator kinetics did not differ across cell populations. Mean (solid line) ± SD (shaded region) calcium response averaged by (A) layer and area, (B) layer, and (C) area. Calcium responses were obtained for each cell by selecting isolated events (those without any other events occurring in the preceding 50 ms or the following 100 ms) and computing the mean event-locked trace (see Materials and Methods, Event detection). D, Responses were well-fit by an exponential decay function; R2 values of the fit for each cell are plotted by layer and area. E, The fit with the lowest R2 value, 0.61 (cell 5 in session 718673398, L2/3 AM; fit in red, data in black). We tested for a relationship between layer, area, and response half-life by fitting a LME model with layer, area, and their interaction as fixed effects and experimental session as a random effect and comparing this to a model without the interaction term; we found no layer × area interaction (likelihood ratio test; χ2(8) = 1.293, p = 0.996). We tested for main effects of layer and area in two further models and likewise found none (layer: χ2(2) = 1.143, p = 0.565; area: χ2(4) = 0.2288, p = 0.994). Download Figure 1-3, TIF file. [file enu-eN-NWR-0280-21-s04.tif]

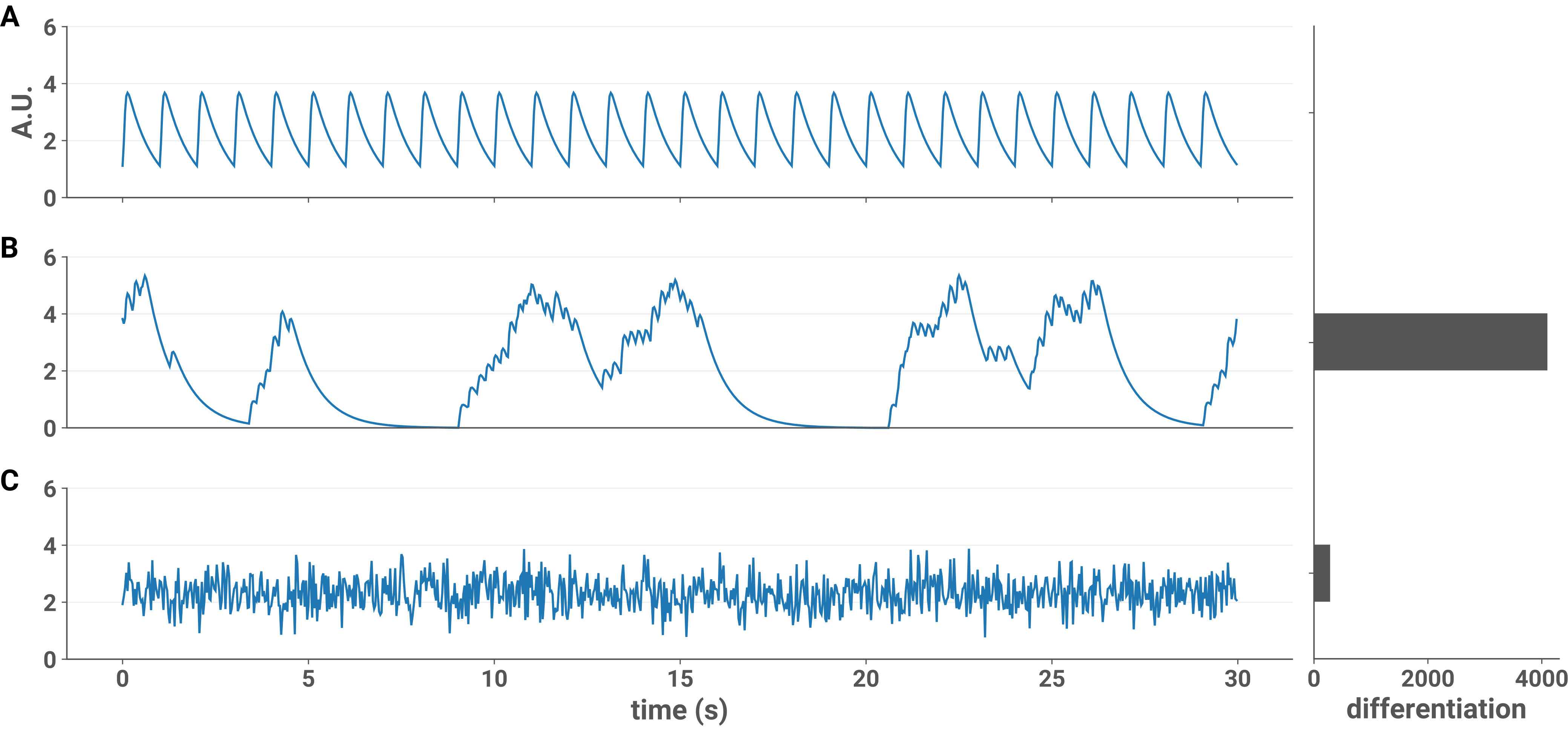

Supplement: Extended Data Figure 2-1 — Differentiation for simulated signals. To illustrate how the ND measure behaves, we generated three artificial signals and computed ND for each. Signals were normalized to have the same energy. A, B, Artificial spike trains were convolved with an idealized GCaMP6f response kernel (difference of exponentials; decay time constant 0.6 s, rise time constant 0.05 s; Chen et al., 2013; Pachitariu et al., 2018) and downsampled to 30 Hz. A, Periodic bursting at 1 Hz. Because the period is the same as the window length used in the spectral estimation step (Fig. 2A), the estimated spectrum of each window is identical, and differentiation is zero. B, An irregular firing pattern has high differentiation. C, Gaussian noise. The theoretical spectrum is identical for each window, but differentiation is nonzero due to the spectral estimation error resulting from the finite window length. Download Figure 2-1, TIF file. [file enu-eN-NWR-0280-21-s05.tif]

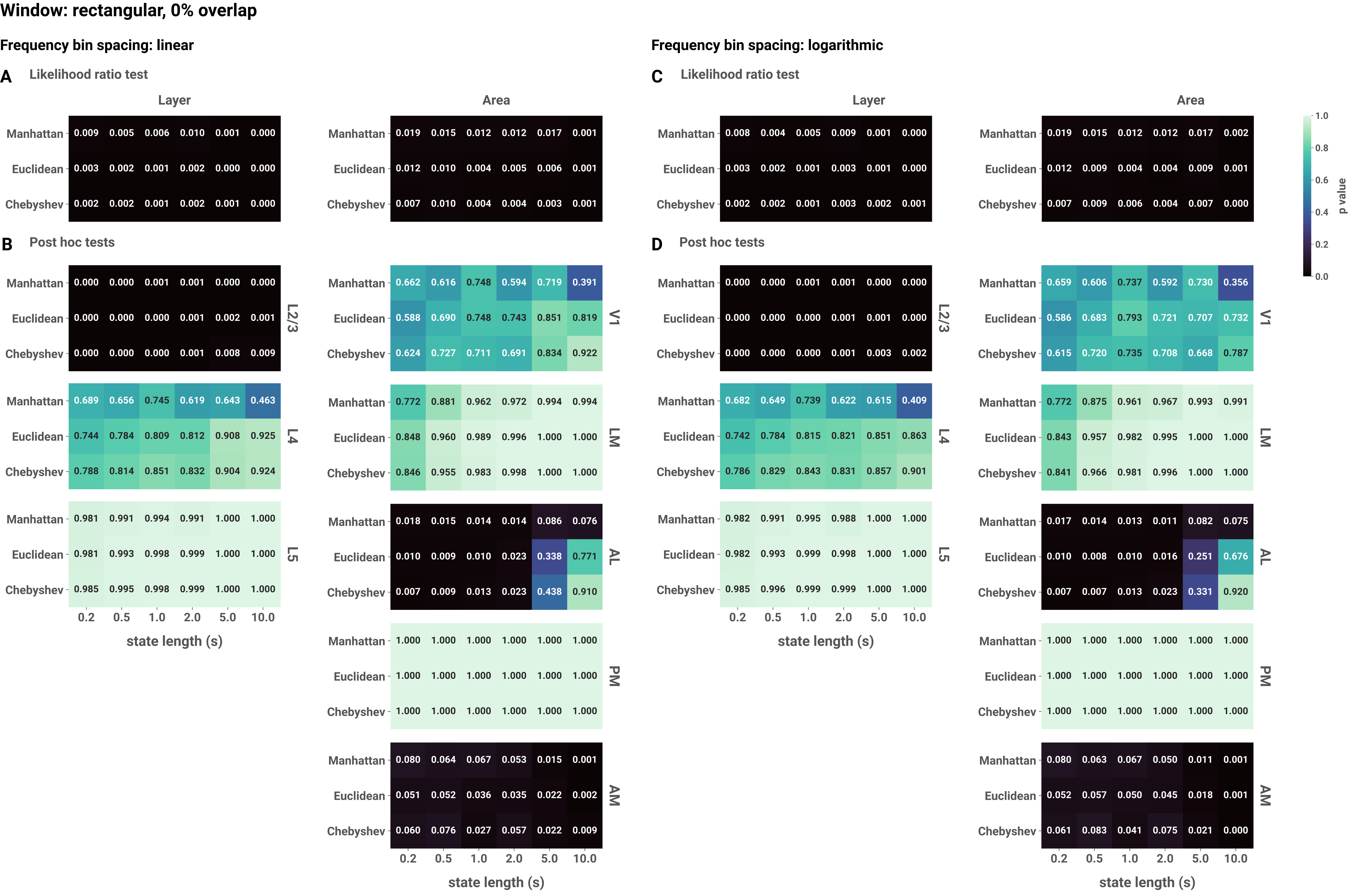

Supplement: Extended Data Figure 3-2 — Sensitivity analysis of main ND results. We investigated the sensitivity of our results to changes in various parameters of the ND calculation. We systematically varied (1) the distance metric used to compare population state vectors (vertical axis of heatmaps); (2) the length of the window that defines a single state, in which the spectrum is estimated (horizontal axis of heatmaps); (3) the spacing of the frequency bins in the estimated spectrum (linear, A, B; logarithmic, C, D); and (4) the window type and amount of overlap used in estimating the spectra across the stimulus presentation (shown in the following two figures). For each combination of these parameters, we computed ND values and performed the same statistical analysis as described in the main text. Each cell in the heatmaps in A, C shows the p value of the likelihood ratio test for the stimulus category × layer interaction (left) and stimulus category × area interaction (right); cells in B, D show p values for the associated post hoc tests. The results reported in the main text correspond to the second row and third column of the heatmaps in A, B. For nearly all other combinations of parameters, we likewise find that unscrambled stimuli elicit increased ND specifically in L2/3 of AL and AM. Download Figure 3-2, TIF file. [file enu-eN-NWR-0280-21-s07.tif]

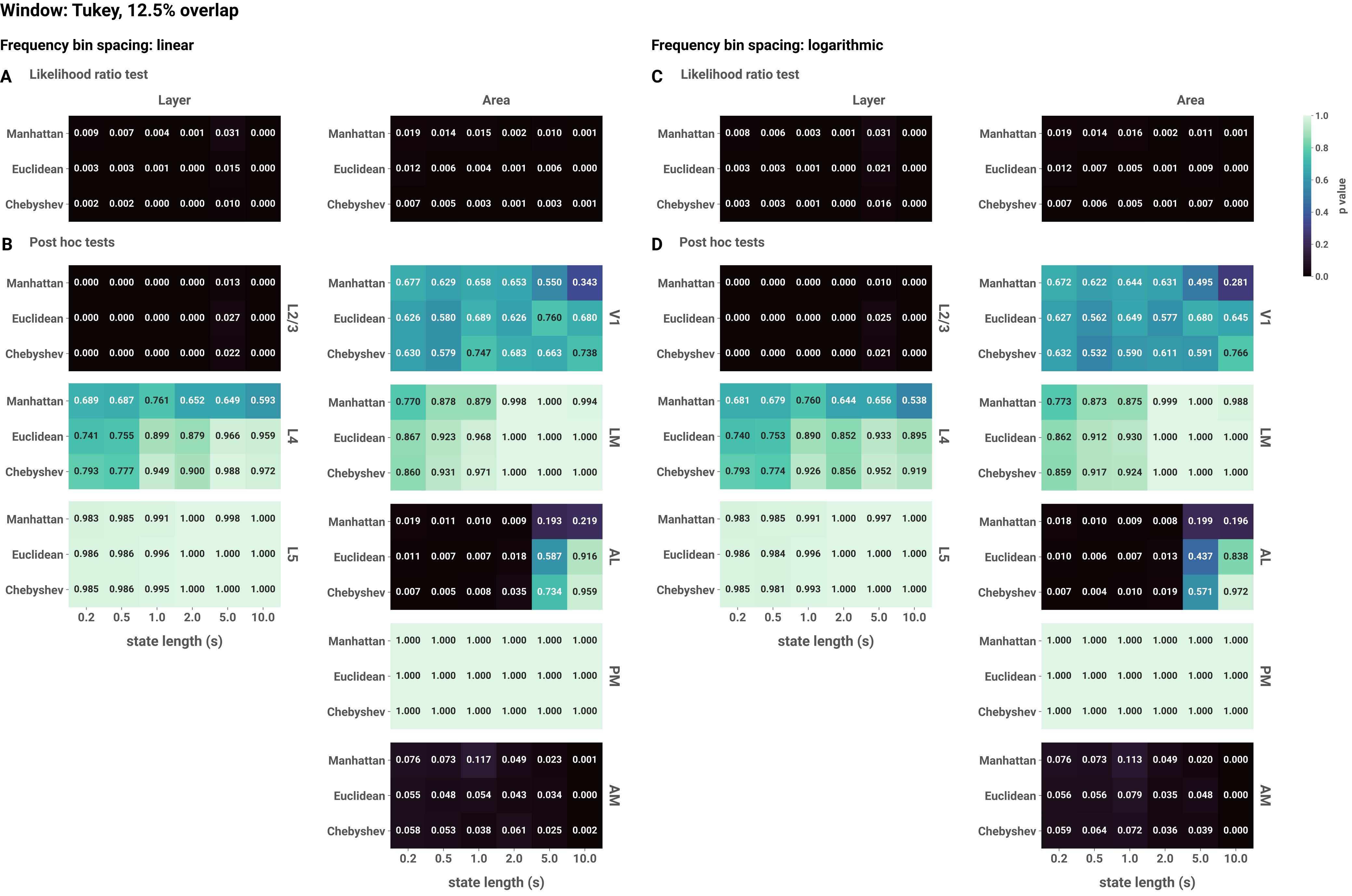

Supplement: Extended Data Figure 3-3 — Sensitivity analysis was performed as described in Extended Data Figure 3-2, except that the time-frequency analysis step of computing ND (Fig. 2A) was performed using a Tukey window with 12.5% overlap. Download Figure 3-3, TIF file. [file enu-eN-NWR-0280-21-s08.tif]

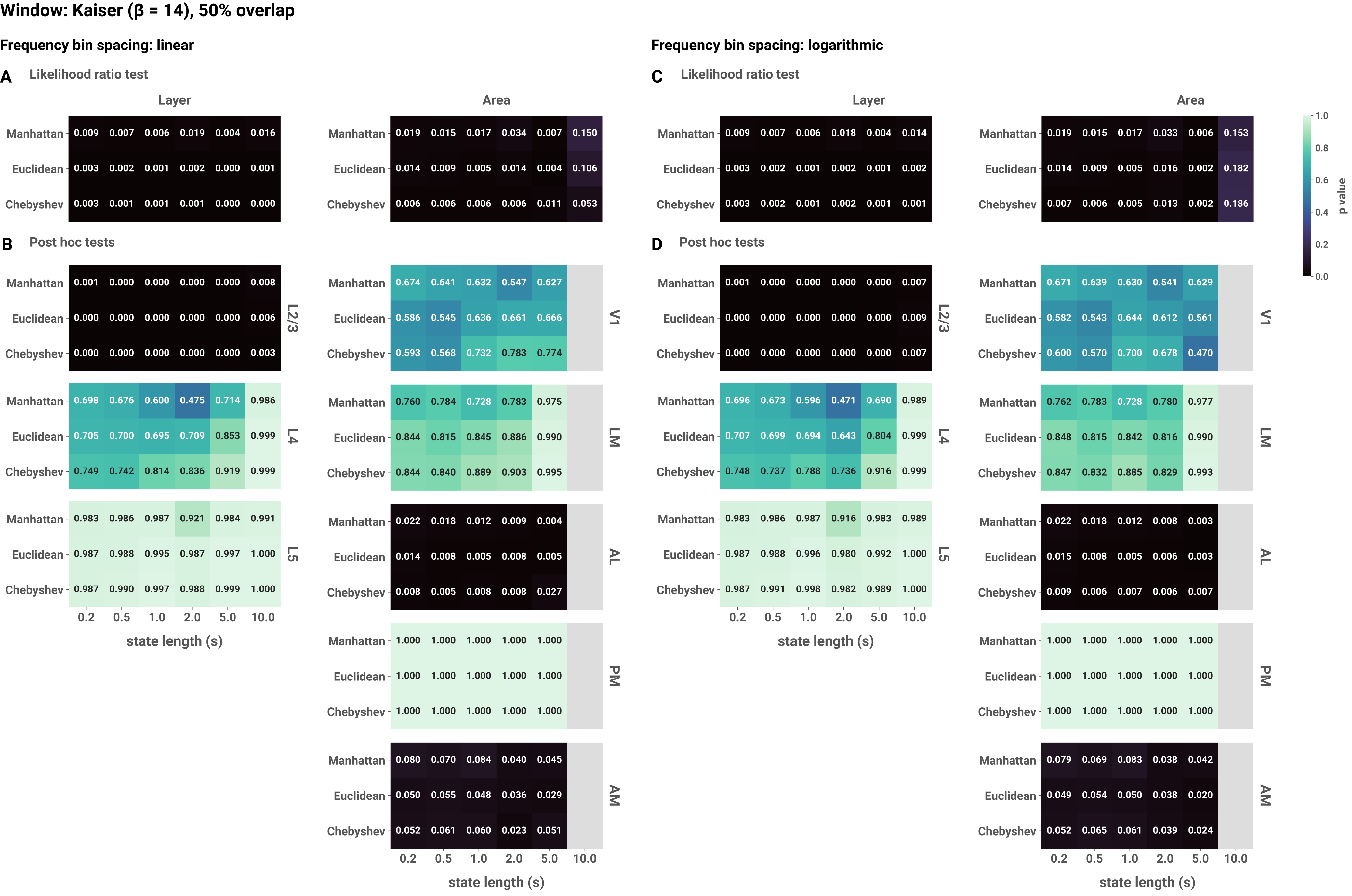

Supplement: Extended Data Figure 3-4 — Sensitivity analysis was performed as described in Extended Data Figure 3-2, except with a Kaiser window (β = 14) with 50% overlap. Download Figure 3-4, TIF file. [file enu-eN-NWR-0280-21-s09.tif]

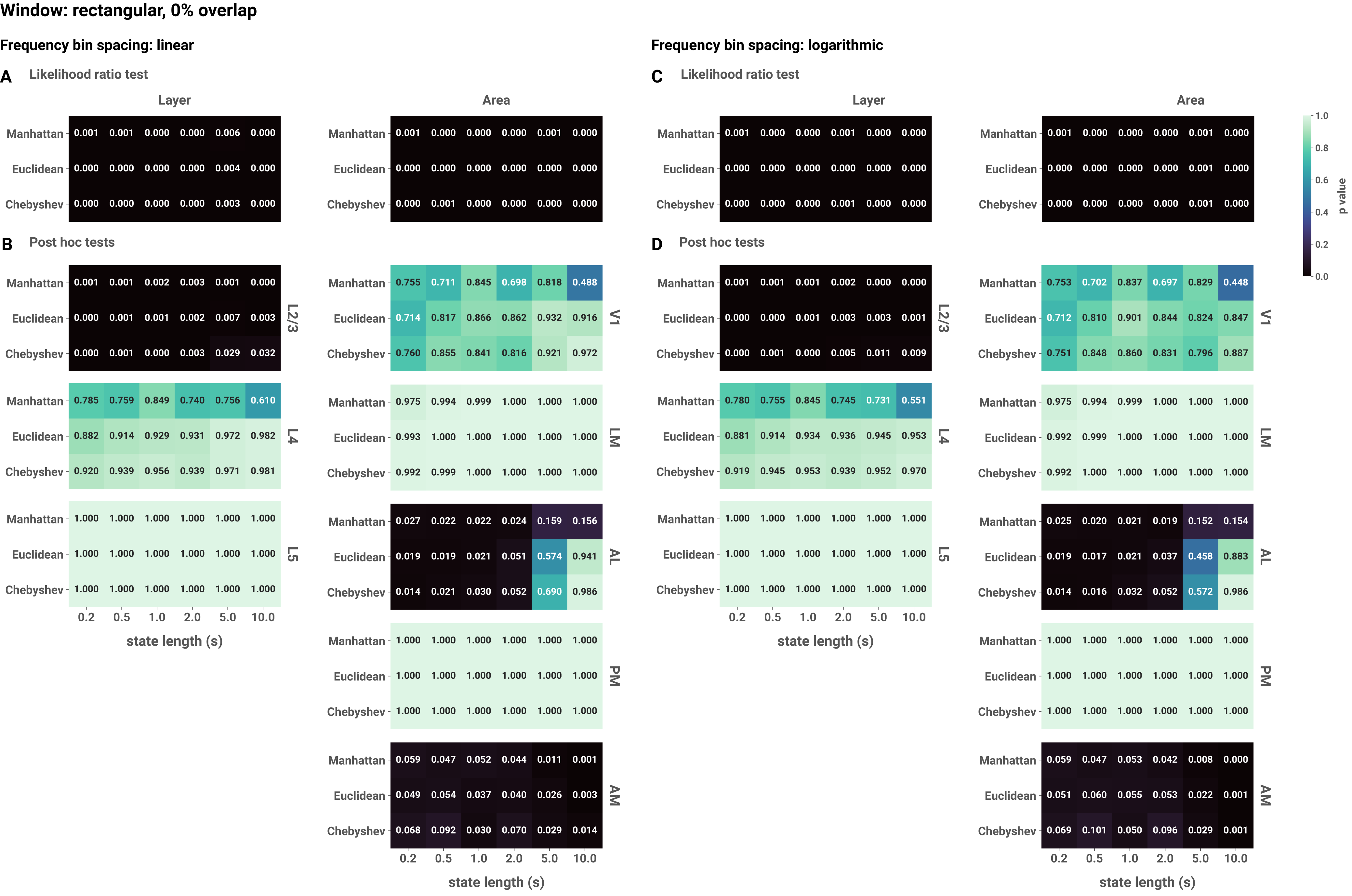

Supplement: Extended Data Figure 3-5 — Sensitivity analysis for LME models including arousal variables (locomotion and pupil diameter) as covariates. Consistent with results from the simpler models, L2/3 of AL and AM emerge as the cell populations in which ND is greater for unscrambled versus scrambled stimuli for nearly all parameter combinations. Download Figure 3-5, TIF file. [file enu-eN-NWR-0280-21-s10.tif]

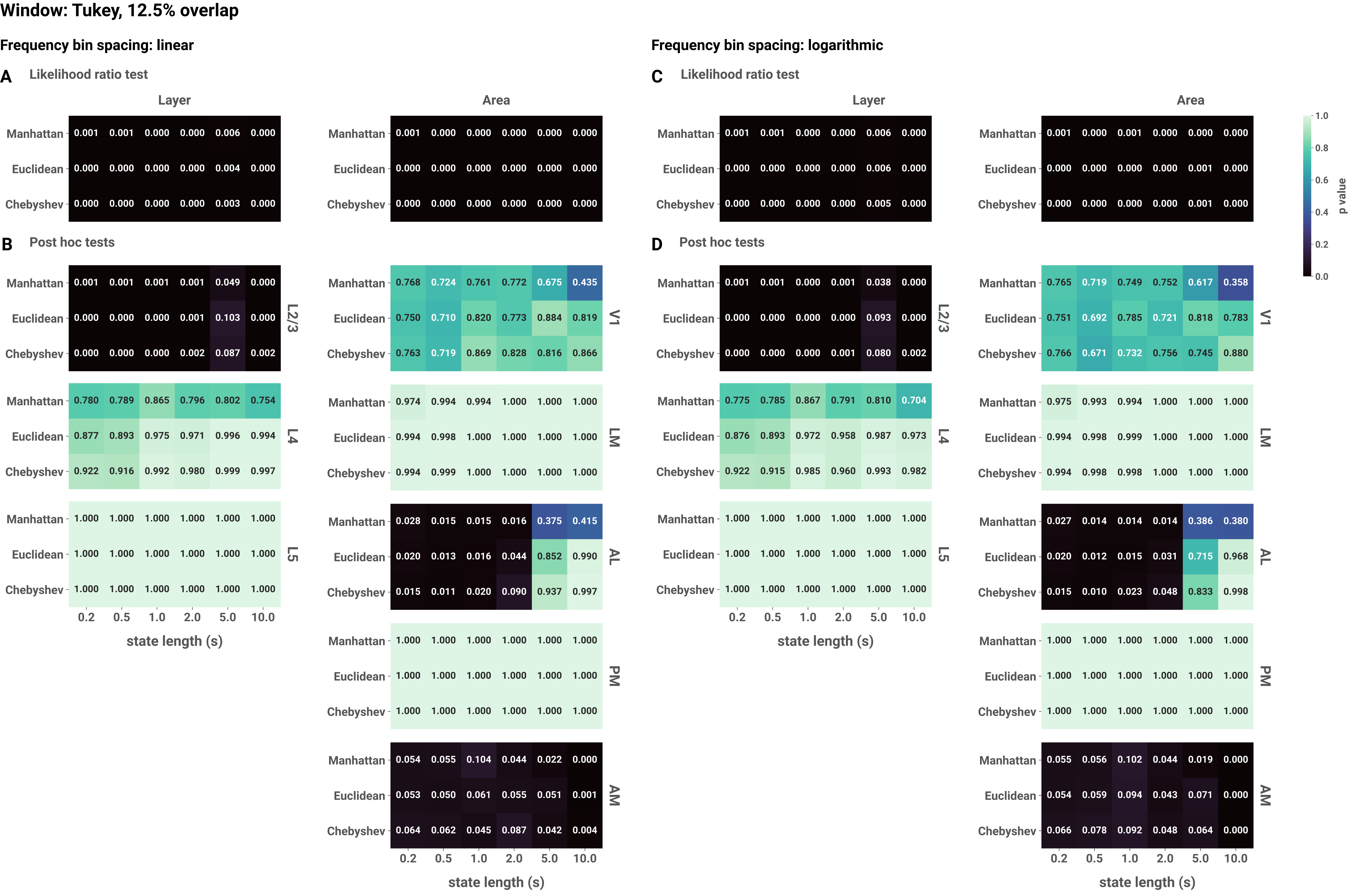

Supplement: Extended Data Figure 3-6 — Sensitivity analysis as in Extended Data Figure 3-5, using a Tukey window with 12.5% overlap. Download Figure 3-6, TIF file. [file enu-eN-NWR-0280-21-s11.tif]

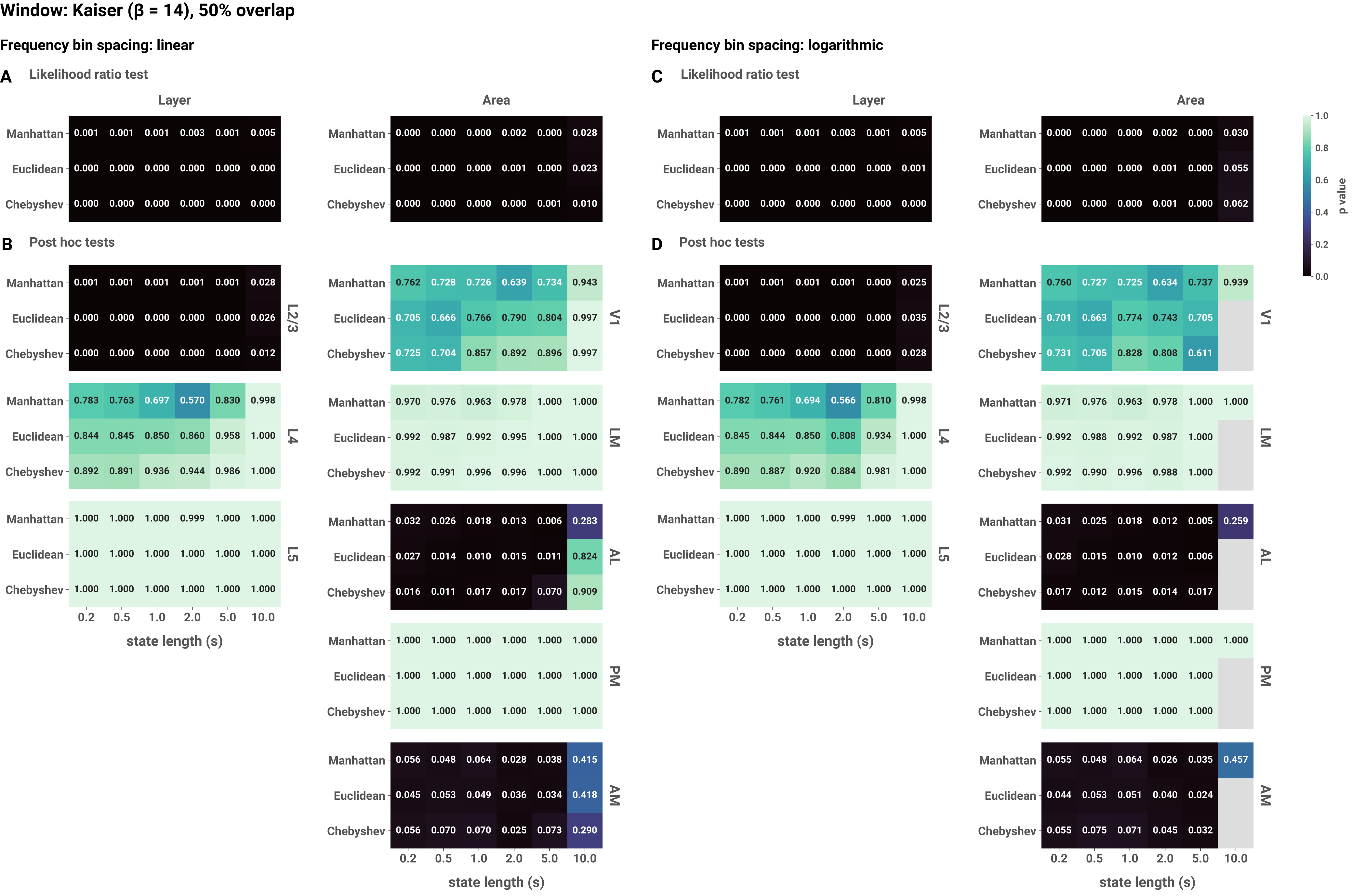

Supplement: Extended Data Figure 3-7 — Sensitivity analysis as in Extended Data Figure 3-5, using a Kaiser window (β = 14) with 50% overlap. Download Figure 3-7, TIF file. [file enu-eN-NWR-0280-21-s12.tif]

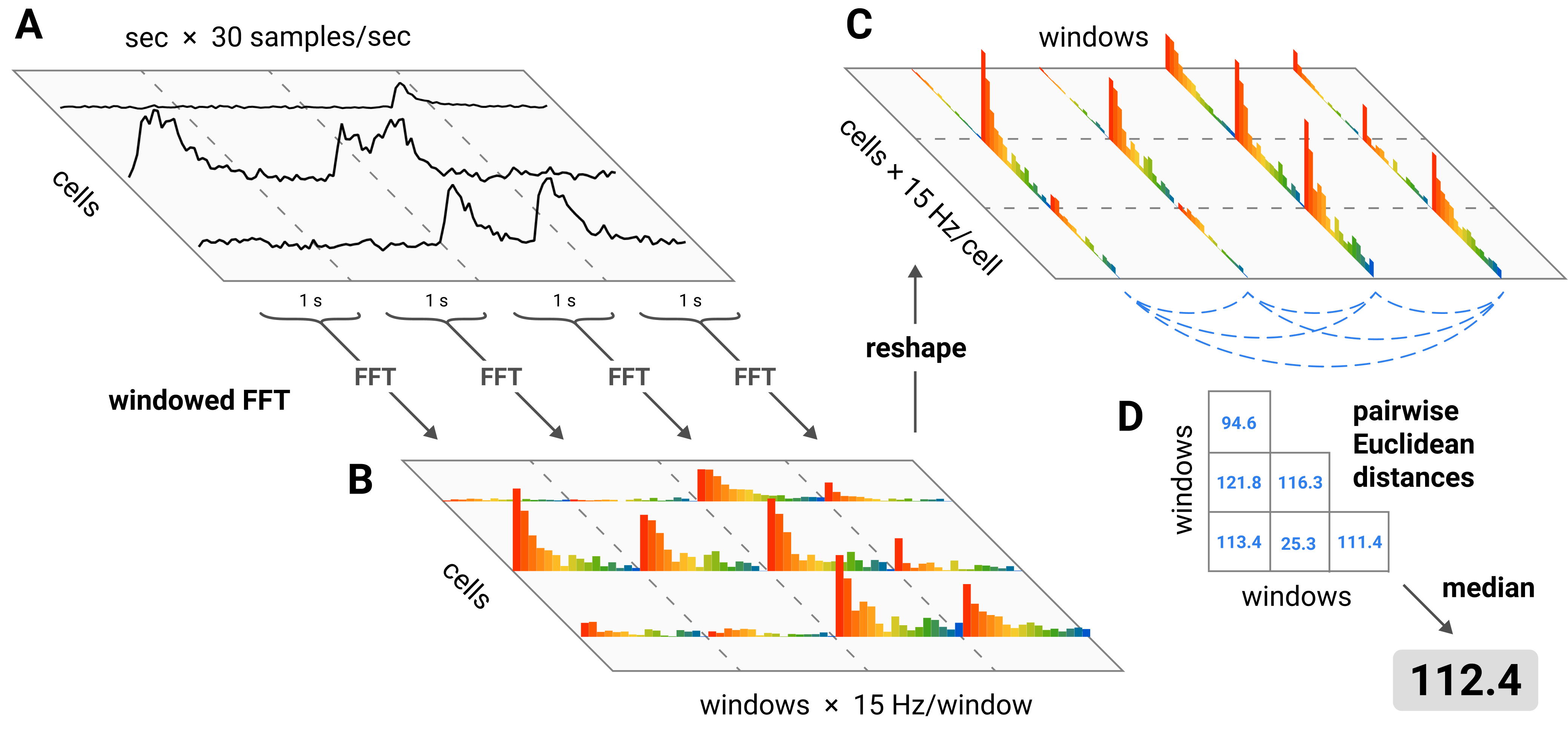

Supplement: Extended Data Figure 7-2 — Within-category differences in ND versus within-category differences in decoding performance, by layer and area. Top, Cohen’s d for pairwise mean differences in ND among naturalistic stimuli without jump cuts. Bottom, Cohen’s d for pairwise mean differences in stimulus identity decoding performance. For each session, we trained a linear discriminant analysis classifier using only responses to these five stimuli; classification performance was evaluated as the mean fivefold cross-validated F1 score for each stimulus (see Materials and Methods, Decoding analyses). Download Figure 7-2, TIF file. [file enu-eN-NWR-0280-21-s17.tif]

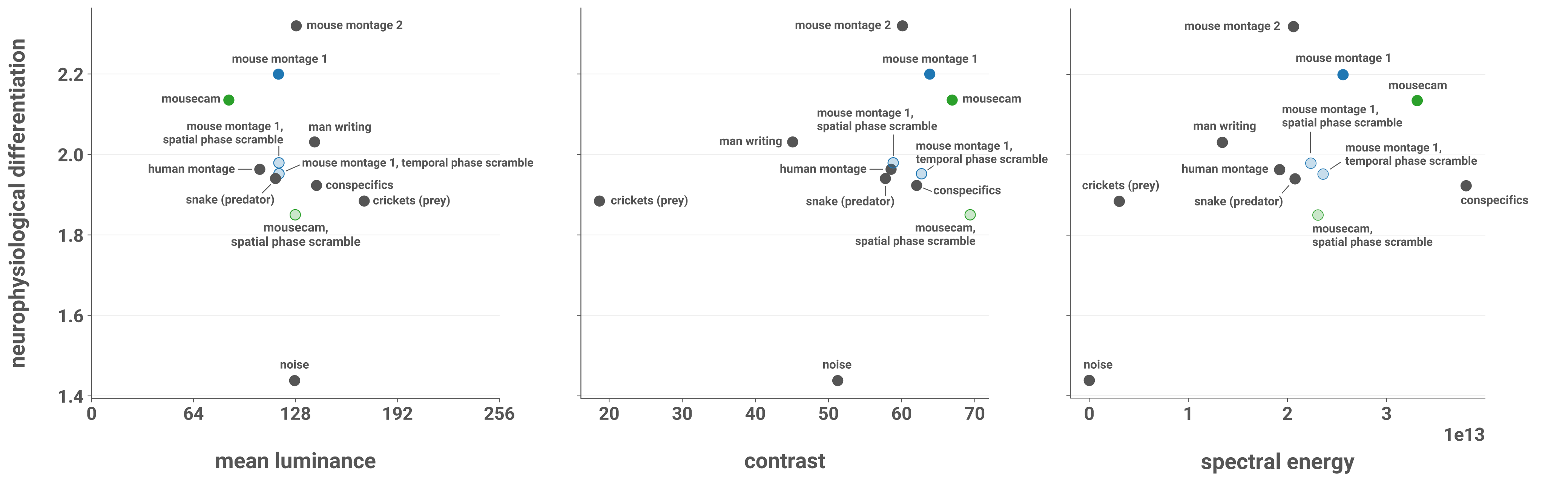

Supplement: Extended Data Figure 8-1 — ND versus low-level stimulus characteristics. ND is plotted against the mean luminance, contrast, and spectral energy of the stimuli. Mean luminance was computed as the average pixel intensity. Contrast was calculated as the SD of pixel intensities. Spectral energy of the blurred stimuli was computed as the sum of the energy spectral density of each pixel’s intensity timeseries after removing the DC component. Download Figure 8-1, TIF file. [file enu-eN-NWR-0280-21-s18.tif]
